# Glycosylated extracellular vesicles drive a metabolic interplay between gastric cancer cells and adipocytes

**DOI:** 10.20517/evcna.2026.16

**Published:** 2026-07-06

**Authors:** Cátia C Ramos, Oihane E Albóniga, Adriana R Rodrigues, Clara Garcia-Vallicrosa, Joana Peixoto, Rui Fernandes, Bruno Cavadas, Alexandra M Gouveia, Celso A Reis, Juan M Falcon-Pérez, Daniela Freitas

**Affiliations:** ^1^i3S - Instituto de Investigação e Inovação em Saúde, Universidade do Porto, Porto 4200, Portugal.; ^2^IPATIMUP - Instituto de Patologia e Imunologia Molecular da Universidade do Porto, Porto 4200, Portugal.; ^3^ICBAS - Instituto de Ciências Biomédicas Abel Salazar, Universidade do Porto, Porto 4050, Portugal.; ^4^Metabolomics Platform, CICbioGUNE-BRTA, Derio 48160, Spain.; ^5^Departamento de Biomedicina, Unidade de Biologia Experimental, Faculdade de Medicina do Porto, Porto 4200, Portugal.; ^6^Faculdade de Ciências da Nutrição e Alimentação, Universidade do Porto, Porto 4200, Portugal.; ^7^Exosomes Laboratory, CIC bioGUNE-BRTA, CIBERehd, Derio 48160, Spain.; ^8^Faculdade de Medicina do Porto, Porto 4200, Portugal.; ^9^IKERBASQUE Research Foundation, Bilbao 48009, Spain.

**Keywords:** Cancer, adipocytes, glycosylation, Sialyl-Tn, extracellular vesicles, metabolomics, fatty acid oxidation, browning

## Abstract

**Aim:** Tumor cells and extracellular vesicles (EVs) dynamically interact with adipocytes, contributing to tumor progression and systemic metabolic dysfunctions. While the role of glycosylation in this process remains unclear, glycans facilitate communication between tumors and their surrounding environment. Here, we aimed to elucidate the pivotal role of sialyl-Tn (STn), a tumor-associated glycan, in driving a bidirectional metabolic reprogramming between gastric cancer cells and adipocytes.

**Methods:** EVs were isolated from gastric cancer cells with distinct glycosylation profiles by ultracentrifugation and characterized by transmission electron microscopy, nanoparticle tracking analysis, and western blotting. Flow cytometry was used to quantify EV uptake by adipocytes. EV-mediated adipocyte browning and lipolysis were measured by real-time quantitative polymerase chain reaction, glycerol and fatty acid release, perilipin immunofluorescence, and western blotting analysis of lipid synthesis and hydrolysis enzymes. The reciprocal effects of beige adipocytes on tumor cells were evaluated using fatty acid uptake assays, metabolomics, RNA sequencing, Seahorse metabolic flux, organelle number, proliferation, and migration assays.

**Results:** STn-positive gastric cancer cells and EVs induced white-into-beige transdifferentiation. These cells exhibited an enhanced capacity to release glycerol and fatty acids, which were predominantly taken up by gastric cancer cells displaying STn. As a feedback mechanism, gastric cancer cells shift their metabolism towards fatty acid oxidation, concomitant with increased mitochondrial and endosomal biogenesis, and enhanced motility.

**Conclusion:** Our findings reveal a key role for the tumor-associated glycan STn in metabolic reprogramming of adipocytes by tumor cells and EVs, opening new avenues for targeted therapies in cancer patients experiencing metabolic dysfunctions, such as cancer cachexia.

## INTRODUCTION

Extracellular vesicles (EVs) released by tumor cells play a crucial role in facilitating communication between cells^[1,2]^. By carrying a diverse array of proteins, lipids, nucleic acids, and glycans, tumor-derived EVs significantly modulate the phenotype and function of recipient cells, thereby driving tumor progression and remodeling the tumor microenvironment^[3-5]^.

Within adipose tissue, adipocytes have emerged as key metabolic partners that actively sustain tumor growth. A growing body of evidence indicates that tumor cells and adipocytes engage in dynamic EV-mediated crosstalk that further supports cancer progression^[5,6]^. Adipocytes are endocrine cells that secrete adipokines, pro-inflammatory cytokines, lipids, and EVs, which can modulate tumor cell proliferation, invasion, angiogenesis, and therapy resistance^[6,7]^. Notably, distinct adipocyte subtypes exhibit distinct metabolic profiles that can differentially affect tumor behavior. White adipocytes primarily store energy as lipids^[8]^ and help regulate metabolic homeostasis^[9]^. In response to tumor-derived signals, white adipocytes can transdifferentiate into beige-like cells. These cells are characterized by increased lipolysis and expression of browning markers, including uncoupling protein 1 (UCP1), peroxisome proliferator-activated receptor gamma coactivator 1-alpha (PGC1α), and PR domain containing 16 (PRDM16)^[10–14]^. Beige-like adipocytes function similarly to brown adipocytes, which are less common in adults, by dissipating energy through mitochondrial thermogenesis^[15,16]^. Nevertheless, upon receiving tumor signals, they can undergo a specific program that increases the release of free fatty acids, which serve as metabolic substrates for cancer cells^[17]^.

In gastric cancer, tumor-derived EVs carrying specific miRNAs have been shown to induce adipocyte transdifferentiation^[18-20]^, increase lipolysis^[20]^, and reduce lipid storage^[18,19]^. Conversely, beige-like adipocytes release free fatty acids^[21,22]^, miRNAs^[23,24]^, and EVs^[21,22,25,26]^. These processes thereby enable tumor cells to switch their metabolism towards fatty acid oxidation. The reciprocal tumor-adipocyte crosstalk not only sustains the high energy needs of proliferative cancer cells but also fosters an immunosuppressive and pro-inflammatory niche that favors tumor aggressiveness^[7,27]^. This tumor EV-mediated metabolic reprogramming negatively impacts cancer patients by driving energetic dysfunctions, such as cancer cachexia, that culminate in reduced quality of life, treatment response, and overall survival^[28]^. It is estimated that approximately 60% of patients with gastric cancer will experience cancer cachexia, resulting in an average weight loss of more than 10%^[29]^.

Emerging evidence shows that tumor-derived EVs carry distinct glycan signatures that can modulate EV uptake by recipient cells^[30-32]^ and ultimately alter their biological effects^[33-35]^. Although less studied in EVs, the sialyl-Tn (STn) antigen is highly synthesized across several cancer types, including gastric cancer^[36]^, and has been associated with increased tumor cell motility and invasion^[37,38]^, metastasis formation^[39-42]^, altered cell adhesion^[37,38]^, immune evasion^[43]^, and poor patient prognosis^[44-49]^. Notably, our previous work revealed that gastric cancer-derived EVs are enriched for the STn antigen^[50]^, and that cells overexpressing this glycan could induce unintentional body weight loss in mouse models^[37]^. Nonetheless, the functional role of EV-associated glycans in communication between tumor cells and adipocytes remains largely unknown.

In this study, we show that gastric cancer cells expressing high levels of STn release EVs that trigger the transdifferentiation of white into beige-like adipocytes. This adipocyte remodeling was characterized by increased expression of browning markers, enhanced lipolytic activity, and the release of glycerol and fatty acids. In turn, STn-positive gastric cancer cells could take up released fatty acids and reprogram their metabolism by activating fatty acid oxidation pathways, increasing mitochondrial number, and enhancing migration capacity. Collectively, we uncover a novel role for the tumor-associated *O*-glycan STn in driving a metabolic interplay between tumor cells and adipocytes via EVs, supporting the malignant features of cancer cells. 

## METHODS

### Cell lines and culture conditions

The human gastric cancer cell line MKN45 was obtained from the Japanese Collection of Research Bioresources. MKN45 Mock and ST6 N-acetylgalactosaminide alpha-2,6-sialyltransferase 1 (ST6GalNAc-I) (MST6-I) cell lines were established by stable transfection with the pcDNA3.1 vector, either empty or encoding the full-length human *ST6GALNAC1* gene, as detailed in^[51]^. Cells were cultured in RPMI 1640 Glutamax medium supplemented with HEPES (Biowest, Nuaillé, France) and 10% fetal bovine serum (FBS) (Gibco, Waltham, USA) and maintained at 37 °C in a 5 % CO_2_ atmosphere. For maintenance of transfected cells, the culture medium was supplemented with 0.6 mg/ml geneticin (G418) (Invivogen, San Diego, USA).

The murine preadipocyte cell line 3T3-L1 MBX (CRL-3242**^TM^**) was acquired from the American Type Culture Collection (ATCC). Cells were cultured in high-glucose DMEM medium (PAN-Biotech, Aidenbach, Germany) supplemented with 10% FBS and allowed to reach 100% confluence. Adipocyte differentiation was initiated two days after reaching confluence using a standard combination of 0.5 mM 3-isobutyl-1-methylxanthine (IBMX) (Sigma-Aldrich, Missouri, USA), 10 μg/mL insulin, and 250 nM dexamethasone (Sigma-Aldrich, Missouri, USA) in DMEM/HAM´S F12 (PAN-Biotech, Aidenbach, Germany) supplemented with 10% FBS. Four days later, the differentiation medium was replaced with complete medium containing 10 μg/ml insulin, and the cells were cultured for an additional four days. Fully differentiated adipocytes were maintained in DMEM/HAM´S F12 supplemented with 10% FBS.

To produce the secretome/conditioned medium (CM), tumor cells and adipocytes were washed with phosphate-buffered saline (PBS) and incubated in serum-free RPMI or DMEM/HAM´S F12, respectively. After 72 h, the supernatants were collected and centrifuged at 300 g for 5 min.

White adipocyte browning was induced by exposing mature adipocytes to either tumor cell-derived secretome (2 mL) or EVs (5 µg) for three days. The medium was refreshed every 24 h with fresh secretome or EVs. The resulting adipocytes were thereafter referred to as transdifferentiated or beige-like adipocytes.

### Immunofluorescence

Cells were seeded onto microscope coverslips (VWR, Carnaxide, Portugal) and allowed to reach 80% confluence for tumor cells or full confluence for white adipocytes. White adipocytes underwent transdifferentiation into beige-like adipocytes using the secretome from MKN45 Mock or MST6-I cells. Cells were fixed with 4% paraformaldehyde (Sigma-Aldrich, St. Louis, USA) for 10 min at room temperature (RT). After fixation, tumor cells were incubated overnight at 4 °C with the B72.3 primary antibody [Table 1], while adipocytes were incubated with the anti-perilipin primary antibody [Table 1]. After washing, tumor cells and adipocytes were incubated with the respective secondary antibodies [Table 1]. Nuclei were counterstained with 4',6-diamidino-2-phenylindole (DAPI) (Sigma-Aldrich, St. Louis, USA). Fluorescence images were captured with a Zeiss Imager Z.1 fluorescence microscope (Zeiss) at 200x and 630× magnifications. 

### Isolation of extracellular vesicles

EVs were isolated by ultracentrifugation from MKN45 Mock and MST6-I cells after 72 h of culture under serum-free conditions, as previously described^[51]^. Briefly, the collected medium was centrifuged at 800 g for 5 min, then at 2,000 g for 10 min. The resulting supernatant was filtered under vacuum through a 0.22 μm constant-pore filtration system (Corning, NY, USA). The filtrate was then ultracentrifuged using a Beckman Optima XE 100 centrifuge equipped with an SW70 Ti fixed-angle rotor and polycarbonate bottles with cap assemblies (Beckman Coulter, California, USA). Ultracentrifugation was performed at 100,000 g for 18 h at 4 °C. Following this, a washing step with 0.9% NaCl solution (Wells, Porto, Portugal) was performed at 100,000 g for 3 h. The final EV pellet was resuspended in 0.9% NaCl solution.

### Nanoparticle tracking analysis

EV size distribution and concentration were determined using a NanoSight NS300 (Malvern Technologies, Malvern, UK), according to the manufacturer’s instructions. The system is equipped with a 488 nm blue laser and a high-sensitivity scientific sCMOS camera. EV samples were diluted in 0.9% NaCl (Wells, Porto, Portugal). Three 60-s videos were recorded per sample, each capturing 749 frames, with the camera level set between 14 and 15. The temperature was maintained between 22.6 °C and 25 °C, and the number of particles per frame ranged from 19.6 to 80.7. Video data were analyzed using NTA software (version 3.2.16). At least four biological replicates were performed, and results are presented as mean ± standard error of the mean (SEM). Student's t-test was used for statistical analysis.

### Transmission electron microscopy

EV morphology, size, and integrity were further characterized by negative-stain transmission electron microscopy (TEM). EV samples were adsorbed onto Formvar/carbon film-coated nickel mesh grids (FCF200-NI-50, Electron Microscopy Sciences) and stained with 1% uranyl acetate.

In addition, Mock and MST6-I cells were analyzed by TEM after exposure to the secretome from white or beige-like adipocytes to visualize subcellular organelles. For ultrastructural analysis, cells were fixed overnight at 4 °C in a solution containing 2% formaldehyde and 2.5% glutaraldehyde in 0.1 M sodium cacodylate buffer. After washing with 0.1 M sodium cacodylate buffer, cells were embedded in Histogel**^TM^** and post-fixed for 1 h in 1% osmium tetroxide in 0.1 M sodium cacodylate buffer. Cells were then stained with aqueous 1% uranyl acetate for 30 min, dehydrated, and embedded in Embed-812 resin. Ultra-thin sections (70 nm thick) were obtained using an RMC Ultramicrotome equipped with Diatome diamond knives (PowerTome, USA), mounted on 200 mesh copper grids (FCF200-NI-50, Electron Microscopy Sciences), and contrasted with uranyl acetate and 3% lead citrate for 5 min each. For each condition, 15 cells were examined, and at least 5 images were acquired per cell. Quantification of mitochondria, lipid droplets, Golgi apparatus, and endosomes was performed using ImageJ. Results are shown as mean ± SEM, and statistical significance was evaluated by One-way analysis of variance (ANOVA).

Digital images of EVs and tumor cells were acquired using a JEOL JEM-1400 transmission electron microscope (JEOL, Tokyo, Japan) equipped with a PHURONA CCD camera (JEOL, Tokyo, Japan).

### Protein quantification

Protein extracts from cell lysates or EVs were prepared in RIPA buffer supplemented with 10 % PhosSTOP**^TM^** and 4% Complete**^TM^** Protease Inhibitor Cocktail (Roche, Basel). Protein content was quantified using the Pierce**^TM^** BCA Protein Assay kit or the Pierce**^TM^** microBCA Assay kit (Thermo Fisher Scientific, Waltham, USA). Absorbance was measured at 562 nm using Gen5.11 software on a Synergy Mx microplate reader. 

### Western blot

Total protein extracts were separated by molecular weight using 10% sodium dodecyl sulfate-polyacrylamide gel electrophoresis (SDS-PAGE) and then transferred to a nitrocellulose membrane (GE Healthcare, UK). After blocking, membranes were incubated overnight at 4 °C with the primary antibodies listed in Table 1. Actin was used as a loading control. After secondary antibody incubation, membranes were developed using Clarity^TM^ Western ECL Substrate (BioRad, California, USA). Chemiluminescence signals were detected and imaged with the ChemiDoc Imaging System (BioRad, California, USA). For some antibodies, band intensities were quantified using Image Lab software. Acetyl-CoA carboxylase (ACC) and hormone-sensitive lipase (HSL) expression levels were normalized to Actin. Phosphorylated HSL levels were normalized to both Actin and total HSL. Two independent experiments were performed, each with one technical replicates each. Results are shown as mean ± SEM. One-way ANOVA was used for statistical analysis.

### Antibodies

**Table 1:** List of antibodies

**Primary antibody**	**Clone**	**Dilution**	**Source**	**Secondary antibody**	**Dilution**	**Source**	**Application**
STn	B72.3	1:3	[52]	Anti-mouse IgG1A/Anti-mouse conjugated to Alexa Fluor 488	1:25,000 / 1:500	Jackson ImmunoResearch	WB/IF
Alix	3A9	1:2,000	Cell Signaling Technology	Anti-mouse	1:5,000	Jackson ImmunoResearch	WB
HSP70	Hsp70A-1	1:1,000	System Biosciences	Anti-rabbit	1:25,000	Jackson ImmunoResearch	WB
Actin	13E5	1:1,000	Cell Signaling Technology	Anti-rabbit	1:25,000	Jackson ImmunoResearch	WB
Syntenin-1	S-31	1:200	Santa Cruz Biotechnology	Anti-mouse	1:5,000	Jackson ImmunoResearch	WB
CD9	C-4	1:200	Santa Cruz Biotechnology	Anti-mouse	1:5,000	Jackson ImmunoResearch	WB
CD81	1.3.3.22	1:200	Santa Cruz Biotechnology	Anti-mouse	1:5,000	Jackson ImmunoResearch	WB
Cytochrome C	7H8	1:200	Santa Cruz Biotechnology	Anti-mouse	1:5,000	Jackson ImmunoResearch	WB
ACC	C83B10	1:1,000	Cell Signaling Technology	Anti-rabbit	1:25,000	Jackson ImmunoResearch	WB
HSL	4107	1:1,000	Cell Signaling Technology	Anti-rabbit	1:25,000	Jackson ImmunoResearch	WB
Phospho-HSL (Ser660) (p-HSL^660^)	4126	1:1,000	Cell Signaling Technology	Anti-rabbit	1:25,000	Jackson ImmunoResearch	WB
Perilipin	D1D8	1:1,000	Cell Signaling Technology	Anti-rabbit conjugated to Alexa Fluor 594	1:500	Thermo Fisher Scientific	IF

STn: Sialyl-Tn; HSP: heat shock protein 70; ACC: acetyl-CoA carboxylase; CD: HSL: hormone-sensitive lipase; IF: immunofluorescence; WB: western blot.

### DiO staining of EVs

Isolated tumor-derived EVs were labeled with 10 μM of the lipophilic dye DiO (Thermo Fisher Scientific, Waltham, USA). The same amount of DiO without EVs was used as a control. The labeling reaction for EVs and control samples (without EVs) was carried out for 20 min at 37 °C. To remove excess unbound dye, labeled EVs and control samples were ultracentrifuged at 100,000 g for 3 h at 4 °C. The EV pellet was resuspended in 0.9% NaCl. 

### EV uptake assay

Mature 3T3-L1 adipocytes were incubated with DiO-labeled EVs (5 μg, approximately 1 × 10^9^ particles) for 1, 3, 6, and 24 h. After incubation, EV uptake by adipocytes was assessed by flow cytometry on a BD LSRFortessa cytometer. FlowJo software (v10; BD Biosciences) was used to analyze the data. Two independent experiments were performed at 1, 3, and 6 h, and three biological experiments were performed at 24 h. Each independent experiment included two technical replicates. Results were normalized to adipocytes treated with control samples (DiO without EVs) and are presented as mean ± SEM. One-way ANOVA was used for statistical analysis.

### RNA extraction and real-time quantitative polymerase chain reaction

White adipocytes were exposed to either tumor cell-derived secretome (2 mL) or EVs (5 µg and 10 µg) for three days. Total RNA was extracted from adipocyte lysates using NZYol Reagent (NZYTech, Lisbon, Portugal) according to the manufacturer’s instructions. RNA concentration and purity were assessed using a NanoDrop ND-1000 spectrophotometer (NanoDrop Technologies, Inc.). For cDNA synthesis, 3 μg of total RNA was reverse transcribed in a 20μL reaction mixture containing 1 μL of random oligonucleotide primers, 1 μL of 10 mM dNTPs, 4 μL of 5X RT buffer, 1 μL of 100 mM DTT, 1 μL of RNAseOUT**^TM^** (40 U/μL), 1μL of SuperScriptR**^TM^** IV reverse transcriptase (200 U/μL) (Invitrogen, Waltham, USA), and RNase-free water. The resulting cDNA was diluted 1:4 with RNase-free water before quantitative polymerase chain reaction (qPCR).

Real-time quantitative polymerase chain reaction (RT-qPCR) was performed using 2 μL of diluted cDNA, 0.6 μL of each primer (10 μM), 10 μL of SYBR® Green Master Mix (Thermo Fisher Scientific, Waltham, USA), and RNase-free water to a final volume of 20 μL. Reactions were run on a 7500 Fast Real-Time PCR System (Applied Biosystems, Massachusetts, USA). Primer sequences are listed in Table 2. For each gene, relative quantification values were determined, and the housekeeping gene Gapdh (NCBI Gene ID: 14433) was used for normalization. Relative gene expression was calculated using the DeltaDelta CT method [relative quantification = 2^-(target Ct – Gapdh Ct)^]. All conditions were normalized to untreated white adipocytes, and the results are presented as ratios of the target gene to Gapdh. For secretome experiments, at least three and six independent biological replicates were performed for *Prdm16* and *Ucp1*, respectively. For EV experiments, at least two independent biological replicates were performed. For each biological replicate, at least two technical replicates were performed per condition. Results are shown as mean ± SEM. One-way ANOVA was used for statistical analysis. 

**Table 2:** Primer sequences for RT-qPCR assays

**Gene**	**Name**	**Species**	**Forward primer (5’-3’)**	**Reverse primer (5’-3’)**
*Ucp1*	Uncoupling Protein 1	Mouse	ACTGCCACACCTCCAGTCATT	CTTTGCCTCACTCAGGATTGG
*Prdm16*	PR Domain Containing 16	Mouse	TGCTAAGCCTTCACCGTTCT	GAAGTTGAACGGGTGGTGAG
*Gapdh*	Glyceraldehyde-3-phosphate dehydrogenase	Mouse	TCTGACGTGCCGCCTGGAG	TCGCAGGAGACAACCTGGTC

RT-qPCR: Real-time quantitative polymerase chain reaction.

### RNA sequencing analysis

Cells were seeded in 6-well plates for 24 h. Cells were then treated for 24 h with 2 mL of secretome from white and transdifferentiated/beige-like adipocytes [TWAT(Mock and MST6-I)]. After treatment, cells were harvested, and RNA was extracted using the PureLinkTM RNA Mini Kit (Invitrogen), according to the manufacturer's instructions. RNA quality was initially assessed using a NanoDropTM One Spectrophotometer (Thermo Fisher Scientific), and the samples were stored at -20 °C until further analysis. Three biological replicates, each with one technical replicate, were performed. Read quality control was assessed using FastQC. Sequence alignment was performed with STAR v2.7.10a using the human GRCh38 reference (GENCODE v44), and gene counts were generated with STAR. Before alignment, reads were pre-processed with TrimGalore to remove adapter sequences and reads shorter than 35 bp from further analysis. Differential gene expression analysis was conducted in R v4.1.2 using DESeq2 (v1.34), considering genes to be differentially expressed if |log_2_(FoldChange)| ≥ 1.2 or |log_2_(FoldChange)| < 1.2 and an adjusted *P*-value ≤ 0.05.

### Glycerol release quantification

Glycerol release from adipocytes was quantified using the Free Glycerol Reagent (Sigma-Aldrich, St. Louis, USA) according to the manufacturer’s instructions. Mature 3T3-L1 adipocytes were incubated for 3 days with the secretome (2 mL) or EVs (5µg) derived from MKN45 Mock or MST6-I cells to induce transdifferentiation of white-to-beige adipocytes. After treatment, adipocytes were washed with PBS and incubated in fresh serum-free DMEM/HAM´S F12 medium. After 72 h, the medium was collected, centrifuged at 300 g for 5 min, and the supernatant was used for glycerol quantification. Absorbance was measured at 540 nm using Gen5.11 software on the Synergy Mx microplate reader. Two biological experiments were performed, each with three technical replicates. Results are shown as mean ± SEM. One-way ANOVA was used for statistical analysis.

### Fatty acid release quantification

Free fatty acid release was measured using the Free Fatty Acid Assay Kit (Sigma-Aldrich, St. Louis, USA), according to the manufacturer’s protocol. Mature adipocytes were incubated with secretome (2 mL) or EVs (5 µg) from MKN45 Mock or MST6-I cells for 3 days to induce transdifferentiation into beige-like adipocytes. Beige-like adipocytes were washed and cultured in fresh serum-free DMEM/HAM´S F12 medium. After 72 h, the supernatant was collected, centrifuged at 300 g for 5 min, and used for fatty acid quantification. Absorbance was measured at 570 nm using Gen5.11 software (Synergy Mx). At least two independent experiments were performed, each with three technical replicates. Results are shown as mean ± SEM, and statistical analysis was performed using One-way ANOVA.

### Fatty acid transfer by 3T3-L1 adipocytes

To assess fatty acid transfer, mature 3T3-L1 adipocytes were incubated with the MKN45 Mock or MST6-I secretome (2 mL) for 3 days to induce transdifferentiation into beige-like adipocytes. Treated adipocytes were washed twice with PBS and incubated with 5 μM BODIPY**^TM^** FL C16 (Alfagene, Carcavelos, Portugal) in serum-free DMEM/HAM´S F12 medium for 6 h. Adipocytes were then cultured in fresh serum-free DMEM/HAM´S F12 medium for an additional 72 h. The resulting secretome was collected and centrifuged at 300 g for 5 min. This medium was added to MKN45 Mock and MST6-I cells (1 × 10^5^ cells) previously seeded on coverslips. Where indicated, cells were immediately treated with etomoxir (50 μM), as reported in^[22]^. After 24 h, cells were fixed with 4% paraformaldehyde, and nuclei were counterstained with DAPI. Fluorescence images were acquired using a Zeiss Imager Z.1 fluorescence microscope (Zeiss) at 630× magnification. Fluorescence intensity was measured using ImageJ software, and the results are shown as mean ± SEM. Two independent experiments were performed, each with one technical replicate, and statistical significance was determined using an unpaired t-test.

### Fatty acid uptake by tumor cells

Fatty acid uptake by tumor cells was evaluated using the Fatty Acid Uptake Kit (Sigma-Aldrich, St. Louis, USA), according to the manufacturer’s instructions. Briefly, 2 × 10^5^ MKN45 Mock or MST6-I cells were seeded into a 96-well plate and treated with secretome from white or beige-like adipocytes for 24 h. Fatty acid uptake was monitored by measuring fluorescence intensity with the Gen5.11 software on a Synergy Mx microplate reader. Two biological experiments were performed, each with three technical replicates. Results are shown as mean ± SEM. One-way ANOVA was used for statistical analysis.

### Metabolomic analysis

#### Metabolite extraction

Mock and MST6-I gastric cancer cells were either untreated or treated with the secretome from white or beige-like adipocytes (1 mL). A biphasic extraction method was used to extract metabolites from the samples. Briefly, 250 μL of cold methanol: water (50:50, v/v) was added to the cell lysates, followed by 200 μL of chloroform to achieve a final solvent composition of 1:1:2 (MeOH: H_2_O: CHCl_3_). Samples were then mixed for 1 h at 4 °C in a Thermomixer Compact (Eppendorf Iberica S.L.U., Madrid, Spain) operating at 171 g (1,400 rpm). Phase separation was achieved by centrifugation at 18,620 g (14,000 rpm) for 30 min at 4 °C using a Mikro 220R centrifuge (HettichLab, Tuttlingen, Germany). The aqueous (polar) layer was collected, completely evaporated, and reconstituted in 130 μL of acetonitrile (ACN): water (ACN: H_2_O; 60:40, v/v). From this volume, 100 μL were transferred into UHPLC-ToF-MS vials, while 40 μL of each sample were combined to generate a pooled quality control (QC) mixture. To remove potential contaminants during sample preparation, two types of blanks were included: a solvent blank (ACN: H_2_O, 60:40, v/v) and an experimental blank processed identically but without any biological material. 

#### Untargeted metabolomics UHPLC-ToF-MS analysis

Metabolite profiling was conducted using an ultrahigh-performance liquid chromatography (UHPLC, Acquity, Waters Inc., Manchester, UK) system coupled to a hybrid quadrupole-time of flight mass spectrometer (Q-ToF MS, SYNAPT G2, Waters Inc., Manchester, UK), as described in^[53]^. Chromatographic separation was performed by injecting 2 µL of sample onto a BEH Amide column (2.1 × 10 mm, 1.7 µm; Waters Inc., Manchester, UK), maintained at 40 °C, with a flow rate of 0.25 mL/min. For the elution gradient, a binary solvent system was used with phase A (1 mM ammonium formate, 0.5% formic acid in water) and phase B (1 mM ammonium formate, 0.5% formic acid, and 5% water in ACN). The gradient started at 20% phase A, reached 99.9% phase A at minute 9, held for 1 min then returned to the starting conditions by minute 10.1 for re-equilibration, resulting in a total run time of 13 min. Mass spectra were acquired in both positive and negative electrospray ionization modes over the mass range 50-1,200 Da. The cone voltage was set to 25 V, and the capillary voltages to 0.25 kV and 0.50 kV for the positive and negative modes, respectively. Other source parameters remain constant: source temperature at 120 °C, desolvation gas (nitrogen) temperature at 450 °C with a flow of 600 L/h, and cone gas (nitrogen) flow at 5 L/h. A 2 ng/mL leucine-enkephalin solution in H_2_O/ACN/formic acid (49.9/50/0.1) was infused at 10 µL/min as a lock mass, and the signal was measured every 36 s for 0.5 s. Spectral peaks were automatically corrected using MassLynx V4.1 software (Waters Inc., Manchester, UK) based on lock mass data. Blank samples were injected at the beginning and at the end of the analytical sequence. A standard polar mixture spanning the full range of physicochemical properties across the entire chromatogram was used to evaluate mass accuracy and chromatographic performance. QC samples were analyzed at the beginning (after blanks) to assess liquid chromatography (LC) system conditioning, and then every 6 randomized samples to minimize time-related effects. These QC samples were used to assess the reproducibility and stability of the system. Finally, QC samples were also analyzed in data-independent acquisition (DIA) mode to obtain fragmentation information for further metabolite identification. DIA method parameters included mass spectrometry (MS) functions matching previous full scans and tandem mass spectrometry (MS/MS) functions with a mass range of 50 to 1,200 Da, a scan time of 0.2 s, and fixed collision energies of 10, 20, 30, and 40 V. 

#### Data processing and deconvolution

MassLynx V4.1 was first used for general data inspection, including verification of chromatographic stability, mass accuracy, pressure traces, and QC reproducibility. MS-Dial v5.1.230807 (https://systemsomicslab.github.io/compms/msdial/main.html) was used for deconvolution, alignment, and matrix generation, using retention time, mass-to-charge ratio (*m/z), *and area measurements for each detected feature (*m/z* - retention time). MS-Dial parameters were adjusted based on the liquid chromatography-mass spectrometry (LC-MS) performance. Features and ions detected in blank samples were excluded from further analysis. Normalization and filtering steps were carried out in R v4.1.2 (R Core Team, 2021), using median fold-change normalization to minimize variability in analytical handling. Features showing a coefficient of variation above 30% in QC samples were removed. Whenever necessary, MATLAB (The MathWorks, Natick, USA) was used to correct for signal intensity decay across the run sequence using the openly available cluster toolbox v2.0 (https://github.com/ Biospec/cluster-toolbox-v2.0). The curated dataset was then imported into SIMCA-P v18.0.1 (Umetrics, Umeå, Sweden) for multivariate statistical analysis.

#### Statistical analysis

Data matrices from gastric cancer MKN45 Mock and MST6-I cells, acquired in both ionization modes, were processed in SIMCA-P for multivariate (MVA) statistical analysis. MVA was used to determine differences between Mock and MST6-I cells before and after exposure to the secretome from white or beige-like adipocytes. SIMCA-P was used for unsupervised (Principal Component Analysis, PCA) and supervised (Partial Least Squares Discriminant Analysis, PLS-DA) approaches. PCA was applied to assess system performance, identify general trends, and detect outlier samples. Samples beyond Hotelling’s T2 at the 95% confidence threshold, as well as QC injections, were excluded from subsequent supervised modeling. PLS-DA models were evaluated using the explained variance (R2) and the predicted variance (Q2) values. For variable selection in PLS-DA models, the p-value from a cross-validation analysis of variance (CV-ANOVA) was used. The validated PLS-DA models were used to select variables and subsequent features. Discriminant features were selected based on variable importance on projection (VIP > 1.0) and an absolute *P*(corr) > 0.85.

#### Metabolic feature identification

The selected features were identified based on accurate m/z values for each metabolite and the fragmentation patterns obtained from MS/MS analysis. Identification was achieved by comparing the results with in-house and online databases and commercial standards. The annotation of confidence levels followed the Metabolomics Standards Initiative (MSI) guidelines^[54]^. 

#### Metabolic pathway analysis

Identified metabolites were subjected to enrichment and pathway analyses using Metaboanalyst 6.0, an online platform. The Homo sapiens pathway library from the Kyoto Encyclopedia of Genes and Genomes (KEGG) database served as the reference. These analyses enabled the identification of key metabolic pathways based on pathway impact and adjusted p-values, calculated using the False Discovery Rate (FDR) approach.

### Proliferation assay

Cell proliferation was assessed using the 5-bromo-2’-deoxyuridine (BrdU) Labeling and Detection Kit (Sigma-Aldrich, St. Louis, USA), according to the manufacturer’s instructions. Briefly, 1 × 10^5^ cells/mL were seeded into a 96-well plate. After 24 h, cells were incubated overnight at 37 °C in a 5 % CO_2_ atmosphere with BrdU Label, diluted 1:2,000 in fresh serum-free medium. The following day, cells were fixed and incubated with an anti-BrdU antibody for 1 h at RT, followed by a 30-min incubation with horseradish peroxidase (HRP)-conjugated goat anti-mouse Immunoglobulin G (IgG) secondary antibody. The substrate solution was then added and incubated in the dark at RT for 15 min, after which the reaction was stopped with the provided stop solution. Absorbance was measured at 450 nm. Results were normalized to the negative control (medium only) and to background noise (cells not exposed to BrdU). Three independent biological experiments were performed, each with three technical replicates. Results are shown as mean ± SEM. Statistical significance was determined using One-way ANOVA. 

### Migration assay

Cell migration was evaluated using a wound-healing assay with 3-well silicone inserts (ibidi, Gräfelfing, Germany). Cells (8 × 10^4^ cells per well) were seeded on each side of the insert and allowed to adhere for 24 h. The insert was then removed to create a defined gap, and detached cells were washed with PBS. Fresh secretome (1 mL from either white or beige-like adipocytes) was added. After 24 h, the medium was replaced with RPMI medium, and cells were monitored for wound closure over 48 h. The cell-free area was quantified using ImageJ by measuring the gap at each time point and normalizing it to the initial free area immediately after insert removal. At least two independent biological experiments, each with four technical replicates per condition, were performed. Results are shown as mean ± SEM, and statistical analysis was performed using two-way ANOVA.

### Seahorse assay

The glycolytic rate of MKN45 Mock and MST6-I cells was measured using the Seahorse XF Glycolytic Rate Assay Kit (Agilent 103344-100) on a Seahorse XFe24 Analyzer (Agilent Technologies, California, USA), following the manufacturer’s instructions. Briefly, 15,000 cells per well were plated in a Seahorse XF24 cell culture microplate and allowed to adhere for 24 h. The following day, tumor cells were treated with 1 mL of secretomes derived from white or beige-like adipocytes and incubated for an additional 24 h. Simultaneously, the Seahorse sensor cartridge was hydrated overnight in Seahorse XF Calibrant (Agilent). On the day of the assay, the culture medium was replaced with Seahorse XF RPMI assay medium (103576-100, Agilent) supplemented with 1 mM pyruvate, 2 mM glutamine, and 10 mM glucose. Cells were incubated for 1 h at 37 °C in a non-CO_2_ incubator, with or without etomoxir (50 μM). The mitochondrial complex inhibitor rotenone/antimycin A (0.5 μM) and the glycolysis inhibitor 2-deoxy-D-glucose (2-DG) (50 mM) were sequentially loaded into the injection ports on the sensor cartridge. Oxygen consumption rate (OCR) and extracellular acidification rate (ECAR) were measured three times in each phase, allowing the evaluation of the basal respiration, mitochondrial ATP production, and maximal glycolytic capacity. When indicated, etomoxir (50 μM, as reported in^[22]^; Sigma-Aldrich, St. Louis, USA) was added 24 h after the initial treatment with secretome from white or beige-like adipocytes. At least two biological experiments, each with three technical replicates per condition, were performed. Results are shown as mean ± SEM, and statistical analysis was performed using One-way ANOVA.

## RESULTS

### STn-positive EVs exhibit enhanced internalization by white adipocytes

In this study, we aimed to understand the role of STn in mediating crosstalk between tumor cells and adipocytes. Therefore, we used the glycoengineered MKN45 ST6GalNAc-I (MST6-I) gastric cancer cell line, which overexpresses ST6GalNAc-I and synthesizes the aberrant STn *O*-glycan, and the MKN45 Mock control cell line, which is negative for STn [Figure 1A and B]. EVs were isolated from both MKN45 Mock and MST6-I cell lines and characterized in accordance with the International Society of Extracellular Vesicles (ISEV) guidelines. Western blot analysis confirmed the presence of specific EV markers, including Heat shock protein 70 (HSP70), Syntenin-1, Alix, Actin, tetraspanins CD9 and CD81, and the absence of the cytochrome C marker, indicating enrichment of EVs in our samples [Figure 1C]. We also detected high-molecular-weight glycoproteins carrying STn in EVs derived from MST6-I cells, whereas Mock EVs were negative for STn [Figure 1C]. Nanoparticle tracking analysis (NTA) showed that the isolated EVs displayed a typical particle size distribution characteristic of EVs [Figure 1D and E]. MST6-I cells secreted significantly more EVs than Mock cells [Figure 1E]. Transmission electron microscopy (TEM) further confirmed the canonical cup-shaped EV morphology of the isolated EVs [Figure 1F]. 

**Figure 1:**
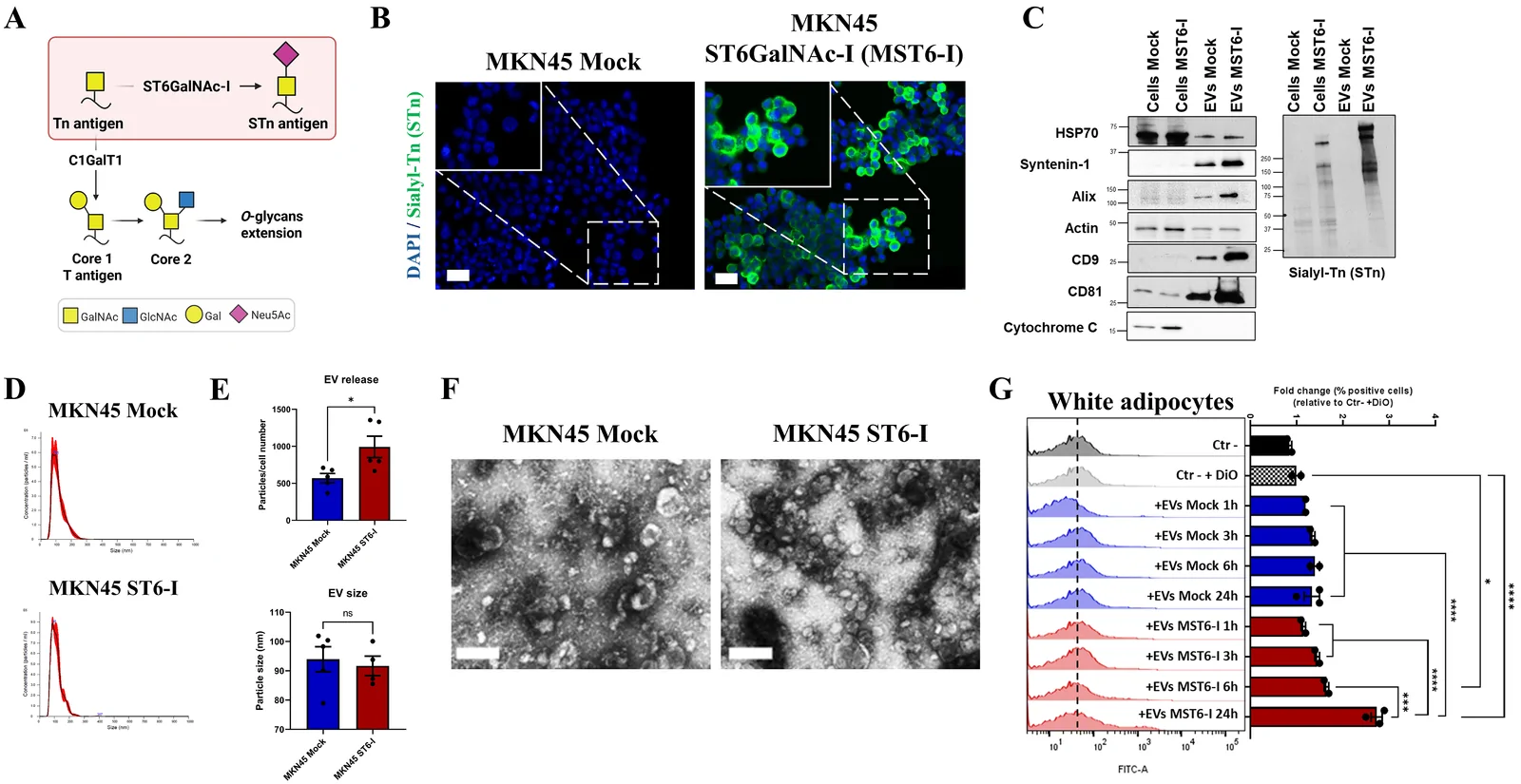
(A) Schematic representation of the biosynthetic pathway of the short *O*-glycan, STn. The MKN45 ST6GalNAc-I (MST6-I) gastric cancer cell line exhibits overexpression of the *ST6GalNAc-I* gene, which codifies the enzyme responsible for STn synthesis. Created in BioRender. Duarte, H. (2026) https://BioRender.com/6xht3c; (B) Immunofluorescence detection of STn antigen in Mock and MST6-I cells (green). Nuclei were counterstained with DAPI (blue). Images were captured at 200× and 630× magnification. Scale bar: 50 µm; (C) Western blot analysis of parental cells and derived EVs for EV markers (HSP70, Syntenin-1, Alix, Actin, CD9, and CD81), mitochondrial marker cytochrome C, and STn antigen. At least two biological replicates were performed for EV markers and three for STn evaluation, each with one technical replicate; (D) Representative Nanoparticle tracking analysis (NTA) histogram image showing the particle-size distribution of Mock and MST6-I-derived EVs; (E) Total particle number and size of EVs released from Mock and MST6-I cells. Results are shown as mean ± SEM. An unpaired *t*-test was used for statistical analysis; (F) TEM images, acquired at 100,000× magnification, of EVs showing their morphology and integrity. Scale bar: 100 nm; (G) Quantification of the uptake of DiO-labeled EVs (5µg) by 3T3-L1 adipocytes after 24 h of incubation. Internalization was assessed by flow cytometry, and results were normalized to control samples (DiO without EVs - Ctr- + DiO). Results are represented as mean ± SEM. One-way ANOVA was used for statistical analysis; unless otherwise indicated, differences were not significant (ns). Biological replicates are represented as individual data points. ns: *P*-value > 0.05; *: *P*-value ≤ 0.05; ***: *P*-value ≤ 0.001; ****: *P*-value ≤ 0.0001. STn: Sialyl-Tn; DAPI: 4',6-diamidino-2-phenylindole; EV: extracellular vesicle; TEM: transmission electron microscopy; SEM: standard error of the mean.

We further evaluated the internalization of DiO-labeled EVs by white adipocytes over time. EVs derived from MST6-I cells, characterized by the presence of the short *O*-glycan STn [Figure 1C], showed increased uptake from 1 h to 24 h of adipocyte exposure [Figure 1G], reaching statistical significance at 6 h and 24 h [Figure 1G]. At the tested time points, STn-negative EVs were never internalized by adipocytes [Figure 1G].

### STn-positive gastric cancer cells and their derived EVs promote adipocyte transdifferentiation and lipolytic remodeling

To assess the impact of gastric cancer-derived soluble factors and EVs on white adipocyte phenotype, we treated 3T3-L1 adipocytes with either the whole secretome or isolated EVs from MKN45 Mock and MST6-I cells [Figure 2A]. We observed that exposing adipocytes to the MST6-I secretome significantly upregulated both *Ucp1* and *Prdm16*, compared with untreated or Mock-treated adipocytes (Figure 2B, left panel). To confirm whether this browning-inducing capacity was consistent across other gastric cancer cell lines expressing short *O*-glycans, we treated 3T3-L1 adipocytes with secretomes from SNU-16 cells (STn-positive) and MKN74 cells (STn-negative) [Supplementary Figure 1A]. Consistently, the SNU-16 secretome significantly increased *Ucp1* expression, whereas the MKN74-derived secretome did not induce this effect [Supplementary Figure 1B]. To assess whether this effect was EV-dependent, adipocytes were incubated directly with EVs isolated from Mock and MST6-I cells. MST6-I EVs significantly induced *Ucp1* expression in a dose-dependent manner, whereas no differences were observed after co-culturing adipocytes with Mock-derived EVs (Figure 2B, right panel). MST6-I EVs also enhanced *Prdm16* expression in adipocytes exposed to higher amounts of EVs, whereas Mock EVs had no impact (Figure 2B, right panel). Interestingly, MST6-I-derived EVs induced a time-dependent upregulation of *Ucp1* and *Prdm16* in adipocytes, with a significant increase in *Ucp1 *expression observed at 24 h compared with untreated adipocytes or those treated with Mock EVs [Supplementary Figure 1C]. 

**Figure 2:**
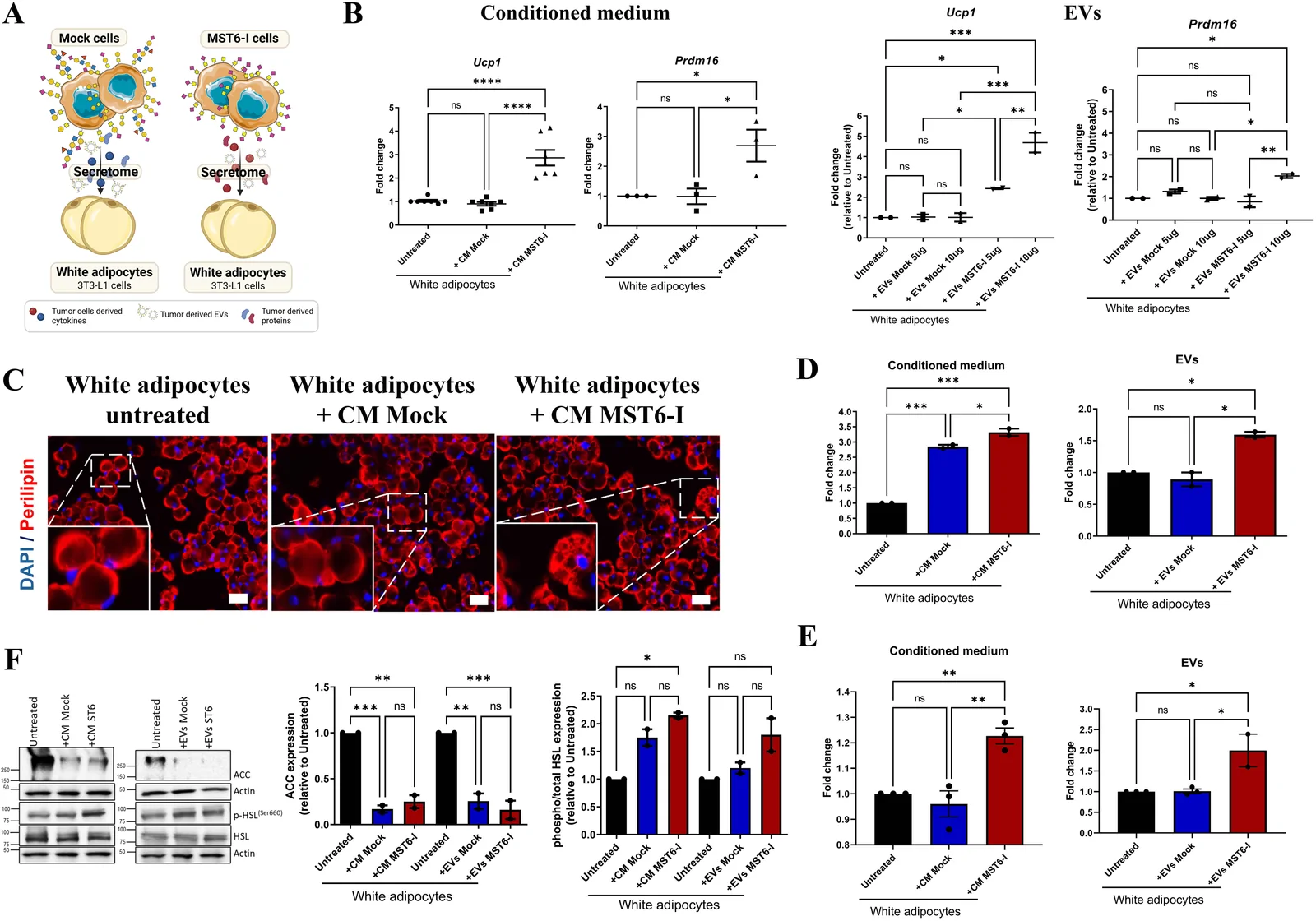
STn-positive gastric cancer-derived EVs and secretome promote browning and lipolysis of 3T3-L1 adipocytes. (A) Schematic representation of the experimental workflow, in which differentiated 3T3-L1 adipocytes were treated with the secretome/CM or isolated EVs from Mock and MST6-I gastric cancer cells. Created in BioRender. Duarte, H. (2026) https://BioRender.com/k39obbd; (B) RT-qPCR analysis of *Ucp1* and *Prdm16* gene expression in 3T3-L1 adipocytes after 24 h of treatment with either the secretome/CM (left panel) or EVs (right panel - 5μ or 10µg) from Mock and MST6-I cells. Results are shown as mean ± SEM, with statistical significance determined by One-way ANOVA; (C) Immunofluorescence images of 3T3-L1 adipocytes (white adipocytes), untreated or treated with the secretome/CM from Mock or MST6-I cells. Lipid droplets were stained for perilipin (red), and nuclei were counterstained with DAPI (blue). Images were acquired at 200× and 630× magnification. Scale bar: 50 µm; (D and E) Quantification of the lipolytic activity by measuring the free glycerol (D) and fatty acid (E) release by 3T3-L1 adipocytes after 24 h of treatment with the secretome/CM or EVs (5μg) from Mock and MST6-I gastric cancer cells. Results are shown as mean ± SEM. One-way ANOVA was used for statistical analysis; (F) Western blot analysis of ACC and phosphorylated HSL expression in untreated 3T3-L1 adipocytes or after being exposed to either the secretome/CM or 5µg of EVs from Mock and MST6-I cells. ACC and HSL expressions were normalized to Actin, and phosphorylated HSL levels were normalized to both Actin and total HSL. Results are shown as mean ± SEM, with statistical significance determined by One-way ANOVA. Biological replicates are represented as individual data points. ns: *P*-value > 0.05; *: *P*-value ≤ 0.05; **: *P*-value ≤ 0.01; ***: *P*-value ≤ 0.001; ****: *P*-value ≤ 0.0001. CM: Conditioned medium; EV: extracellular vesicle; ACC: acetyl-CoA carboxylase; DAPI: 4',6-diamidino-2-phenylindole; RT-qPCR: real-time quantitative polymerase chain reaction; ANOVA: analysis of variance; HSL: hormone-sensitive lipase; SEM: standard error of the mean.

In addition, we observed that the MST6-I secretome induced lipolytic remodeling in adipocytes. Perilipin staining was used to visualize lipid droplet morphology [Figure 2C]. Although single large lipid droplets were often observed across all conditions, they were more common in untreated adipocytes or in those treated with the secretome from Mock cells [Figure 2C]. In contrast, smaller lipid droplets were observed in adipocytes treated with the secretome from MST6-I cells [Figure 2C]. This morphological shift was accompanied by increased levels of glycerol and free fatty acids released from adipocytes, indicating enhanced lipolytic activity upon treatment with the MST6-I secretome or EVs [Figure 2D and E]. While the secretome from Mock cells also induced glycerol release by adipocytes, their EVs alone had no effect [Figure 2D]. Furthermore, neither the secretome nor the EVs from Mock cells induced the release of free fatty acids from adipocytes [Figure 2E]. Western blot analysis further showed that adipocytes treated with the secretome or EVs from Mock or MST6-I cells exhibited reduced levels of acetyl-CoA carboxylase (ACC), a key enzyme in fatty acid synthesis. In addition, increased phosphorylation at Ser-660 of HSL, a marker of lipolysis, was also observed when adipocytes were exposed to the secretome from Mock or MST6-I cells [Figure 2F]. Furthermore, EVs from MST6-I promoted increased expression of the phosphorylated form of HSL (p-HSL 660), compared to untreated adipocytes [Figure 2F]. In contrast, Mock EVs did not show the same effects [Figure 2F].

Collectively, we observed that secreted factors and EVs from MST6-I cells induce a phenotypic change in adipocytes, consistent with browning and lipolytic remodeling of white adipocytes, to a greater extent than either the Mock secretome or derived EVs.

### Beige-like adipocytes trigger metabolic changes in gastric cancer cells

We next assessed the feedback impact of transdifferentiated/beige-like adipocytes on the metabolism of gastric cancer cells. We started by generating the secretome/CM of the beige-like adipocytes by exposing them to either Mock or MST6-I cell secretome [designated as Transdifferentiated White Adipocytes - TWAT(Mock) and Transdifferentiated White Adipocytes - TWAT(MST6-I), respectively; Supplementary Figure 2A]. The secretome produced by beige-like adipocytes [TWAT(Mock) or TWAT(MST6-I)] was then used to treat both Mock and MST6-I tumor cells, and metabolomic analysis was performed [Supplementary Data 1]. We first evaluated the baseline metabolic profiles of Mock and MST6-I cells using multivariate analysis, including PCA, and found that these cells have distinct baseline metabolic profiles [Supplementary Figure 2B]. Furthermore, both cell lines significantly altered their metabolic signatures after exposure to the adipocyte-derived secretome, with treatment conditions exerting a greater influence on metabolic profiles than the inherent baseline differences between the Mock and MST6-I cell lines [Figure 3A and B]. 

**Figure 3:**
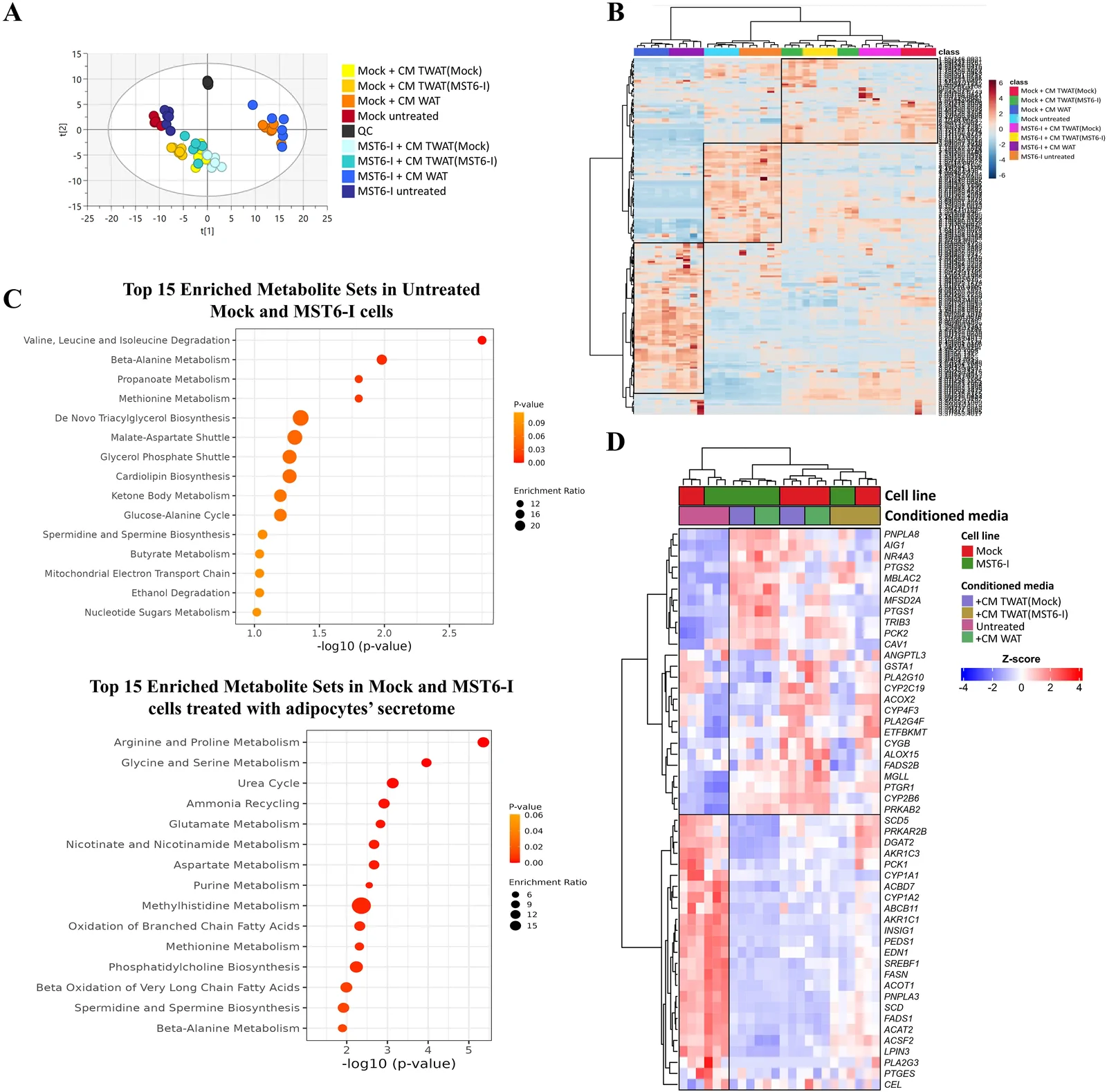
Adipocyte-derived secretome reprograms gastric cancer cell metabolism. (A) PCA scores plot of Mock and MST6-I cells (R2X[1] = 0.339, R2X[2] = 0.136, 2 PCs and Pareto scaling), untreated or treated with the secretome/CM from white or TWAT(Mock) and TWAT(MST6-I) adipocytes; (B) Hierarchical clustering of the metabolomic profiles of Mock and MST6-I cells, untreated or treated with the secretome/CM from white or TWAT(Mock) or TWAT(MST6-I) adipocytes; (C) Pathway analysis of the top 15 enriched metabolite sets identified as upregulated in untreated Mock and MST6-I cells (upper panel) or after exposure to secretome/CM from white or TWAT(Mock) and TWAT(MST6-I) adipocytes (lower panel); (D) Heatmap representation of transcriptomic profiles of Mock and MST6-I cells, either untreated or treated with secretome/CM from white or TWAT(Mock) and TWAT(MST6-I) adipocytes, related to fatty acid metabolic processes. CM: Conditioned medium. PCA: principal component analysis; PC: principal component.

Through hierarchical clustering analysis, we observed that Mock and MST6-I cells treated with the secretome from white adipocytes cluster together and separately from those treated with secretomes from beige-like adipocytes [TWAT(Mock) or TWAT(MST6-I)], which also cluster together [Figure 3B]. This indicates that although Mock and MST6-I cells have distinct baseline metabolisms, exposure to secretomes from white or beige-like adipocytes induces similar metabolic alterations in both cell lines under each condition. We have further identified metabolites in Mock and MST6-I cells, both untreated and after exposure to secretomes from white- or beige-like adipocytes [Supplementary Data 2]. Using the identified metabolites within each cluster in the heatmap in Figure 3B, we determined the top 15 enriched metabolic pathways for untreated and treated Mock and MST6-I cells. Pathway analysis of metabolites upregulated in untreated Mock and MST6-I cells revealed enrichment in valine, leucine, and isoleucine degradation, as well as lipid- and energy-related pathways, including triacylglycerol biosynthesis, the glycerol phosphate shuttle, and ketone body metabolism (Figure 3C, upper panel). In contrast, metabolites identified as upregulated in Mock and MST6-I cells treated with secretomes from white or beige-like adipocytes [TWAT(Mock) or TWAT(MST6-I)] were enriched in amino acid–related pathways and fatty acid oxidation pathways, particularly the oxidation of branched-chain fatty acids and beta oxidation of very long-chain fatty acids (Figure 3C, lower panel). PLS-DA of the metabolic data further confirmed distinct metabolic profiles between Mock and MST6-I cells treated with the secretomes from white or beige-like adipocytes [TWAT(Mock) and TWAT(MST6-I)] [Supplementary Figure 2C]. We observed an increase in biosynthetic pathways involving alanine, aspartate, glutamate, glutathione, glycerophospholipids, and arginine in both Mock and MST6-I cells treated with secretomes from different adipocyte types [white or TWAT(Mock) or TWAT(MST6-I)] compared with the corresponding untreated cells [Supplementary Figure 2D]. Fatty acid metabolism was upregulated in both Mock and MST6-I cells across treatments with secretomes from different adipocyte types, compared with untreated cells, although to a lesser extent. This effect was particularly pronounced in MST6-I cells treated with secretome from TWAT(MST6-I) adipocytes [Supplementary Figure 2D]. Transcriptomic profiling of fatty acid metabolic genes also revealed two distinct clusters that separated the untreated Mock and MST6-I cells from those treated with secretomes from white or TWAT(Mock) or TWAT(MST6-I) adipocytes [Figure 3D]. In particular, untreated cells displayed higher expression of genes involved in fatty acid synthesis (*SDD5*, *FASN*, *SCD*, and *FADS1*), triglyceride metabolism (*DGAT2),* and lipogenesis (*INSIG1*, *SREBF1*). In contrast, both Mock and MST6-I cells treated with secretomes from white or beige-like adipocytes showed increased expression of genes associated with lipid remodeling (*AIG1, PLA2G1, *and* PLA2G4F) *and polyunsaturated fatty acids metabolism (*MFSD2A*, *ACOX2, *and* CYP2C19*)*. *MST6-I cells exhibited increased expression of fatty acid oxidation genes (*ACAD11* and *PRKAB2*), lipid remodeling genes (*PNPLA8*), and fatty acid trafficking genes (CAV1) when treated with secretomes from white or TWAT(MST6-I) adipocytes, compared with Mock cells [Figure 3D].

These findings demonstrate that adipocytes significantly influence the metabolism of gastric cancer cells. We observed that exposure to the secretome from white or beige-like adipocytes induced metabolic reprogramming in tumor cells, which surpassed the inherent differences between Mock and MST6-I cells. Transcriptomic analysis further revealed upregulation of genes involved in lipid remodeling and fatty acid oxidation, particularly in MST6-I cells treated with the secretome from white or beige-like adipocytes.

### Beige-like adipocytes promote fatty acid metabolism and alter the phenotypic features of STn-positive gastric cancer cells

Our previous findings demonstrated that adipocytes induce metabolic reprogramming in tumor cells. To further elucidate how these adipocytes affect fatty acid oxidation in tumor cells, we treated Mock and MST6-I gastric cancer cells with the secretome from white or beige-like adipocytes [TWAT(Mock) and TWAT(MST6-I)] [Figure 4A] and performed a Seahorse analysis. We observed distinct basal metabolic profiles between Mock and MST6-I cells [Supplementary Figure 3A], with untreated Mock cells exhibiting a greater tendency toward higher glycolytic rates than untreated MST6-I cells (Figure 4B; left panel). We observed that inhibition of mitochondrial fatty acid oxidation with etomoxir, a carnitine palmitoyl transferase (CPT1) inhibitor, resulted in a marked increase in basal glycolytic flux in MST6-I cells when they were exposed to the secretome from white or beige-like adipocytes [TWAT(MST6-I); Figure 4B, left panel]. Additionally, when fatty acid oxidation was blocked by etomoxir, MST6-I cells exposed to secretomes from white or TWAT(MST6-I) adipocytes showed a compensatory increase in glycolytic flux (Figure 4B, right panel), demonstrating a preferential reliance on fatty acid oxidation under substrate exposure.

**Figure 4:**
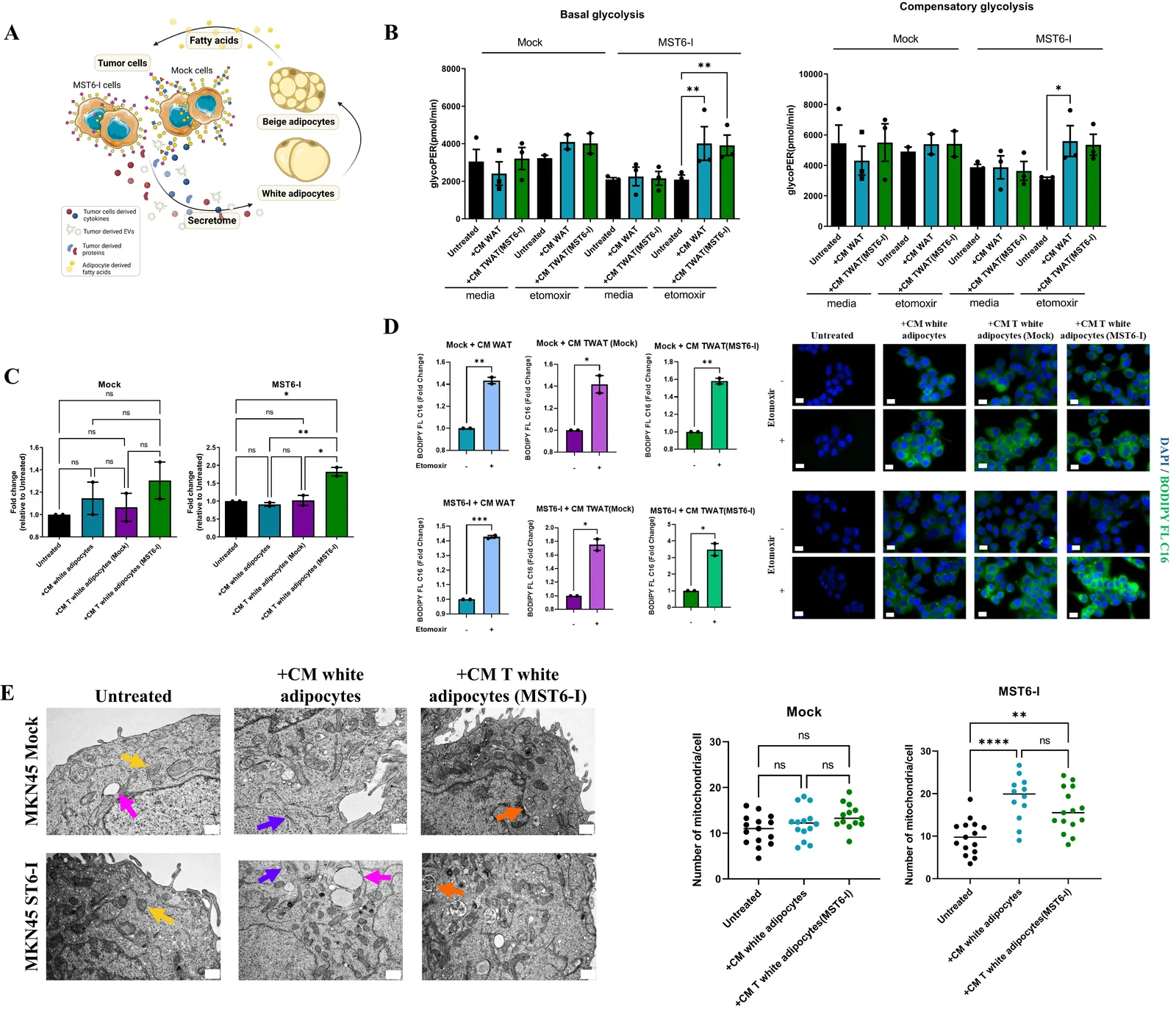
Adipocyte-derived secretome enhances fatty acid metabolism and mitochondria biogenesis in STn-positive gastric cancer cells. (A) Schematic illustration of the workflow used, in which 3T3-L1 adipocytes were treated with Mock or MST6-I secretome/CM to generate transdifferentiated white adipocytes (TWAT)(Mock) or TWAT(MST6-I). The secretome/CM from these adipocytes, enriched in free fatty acids, was used to treat the gastric cancer cells. Created in BioRender. Duarte, H. (2026) https://BioRender.com/iz3w8pf; (B) Seahorse analysis of glycolytic activity in Mock and MST6-I cells treated with secretome/CM from white or transdifferentiated/beige adipocytes [TWAT(MST6-I)]. When indicated, etomoxir was added. Quantification of basal (left panel) and compensatory glycolysis (right panel) is shown as mean ± SEM. One-way ANOVA was used for statistical analysis; unless otherwise indicated, differences were not significant (ns); (C) Quantification of fatty acid uptake by Mock and MST6-I gastric cancer cells relative to untreated controls. Results are shown as mean ± SEM, with statistical significance determined by One-way ANOVA; (D) Fluorescence microscopy images and quantification of fluorescence intensity in Mock (upper panel) and MST6-I (bottom panel) cells, untreated or incubated with secretome/CM from white or transdifferentiated/beige adipocytes pre-labeled with BODIPY FL C16 (green). Where indicated, etomoxir was added. Nuclei were counterstained with DAPI (blue). Images were acquired at 630× magnification. Scale bar: 10 µm. Results are shown as mean ± SEM, with statistical significance determined by an unpaired t-test; (E) Representative electron microscopy images of Mock and MST6-I cells after treatment with white or transdifferentiated/beige [TWAT(MST6-I)] secretome/CM (left panel) and quantification of mitochondrial number per cell (right panel). Arrows indicate mitochondria (yellow), the Golgi apparatus (purple), lipid droplets (pink), and endosomes (orange). Images were acquired at 20,000× magnification. Scale bar: 500 nm. Results are shown as mean ± SEM, with statistical significance determined by One-way ANOVA. Biological replicates are represented as individual data points. ns: *P*-value > 0.05; *: *P*-value ≤ 0.05; **: *P*-value ≤ 0.01; ***: *P*-value ≤ 0.001; ****: *P*-value ≤ 0.0001. CM: Conditioned medium; STn: sialyl-Tn; SEM: standard error of the mean; ANOVA: analysis of variance; DAPI: 4',6-diamidino-2-phenylindole.

Consistent with this metabolic reprogramming, we also observed that MST6-I cells treated with the secretome from TWAT(MST6-I) adipocytes showed significantly higher fatty acid uptake than untreated cells or those treated with secretomes from white or TWAT(Mock) adipocytes [Figure 4C]. In contrast, Mock cells revealed no change in fatty acid uptake [Figure 4C]. To follow the transfer of fatty acids from adipocytes to tumor cells, we pre-labeled adipocytes with BODIPY FL C16, a fluorescent fatty acid analog, and incubated both Mock and MST6-I cells with the secretome from those adipocytes. Fluorescence microscopy images revealed increased fatty acid accumulation in Mock and MST6-I cells incubated with secretomes from white or beige-like adipocytes after etomoxir treatment, particularly in MST6-I cells exposed to the secretome of TWAT(MST6-I) adipocytes [Figure 4D]. Furthermore, MST6-I cells treated with the secretome from both white and TWAT(MST6-I) adipocytes showed a significant increase in mitochondrial content, an effect absent in Mock cells [Figure 4E]. No significant changes were observed in the number of lipid droplets or the Golgi apparatus in either Mock or MST6-I cells [Supplementary Figure 3B and C]. Notably, MST6-I cells exhibited enhanced endosome formation following exposure to the secretome from TWAT(MST6-I) adipocytes [Supplementary Figure 3D], suggesting additional alterations in intracellular trafficking and possibly exosome production.

Following the metabolic alterations induced by secretomes from white or beige-like adipocytes, we evaluated their functional impact on Mock and MST6-I cells. Treatment of both Mock and MST6-I cells with secretomes from different adipocyte types did not affect the proliferation rates of either cell line [Figure 5A]. However, we observed a significant increase in the migration capacity of Mock and MST6-I gastric cancer cells upon exposure to secretomes from both white and TWAT(Mock or MST6-I) adipocytes, with a greater effect in MST6-I cells exposed to the secretome from TWAT(MST6-I) adipocytes [Figure 5B].

**Figure 5:**
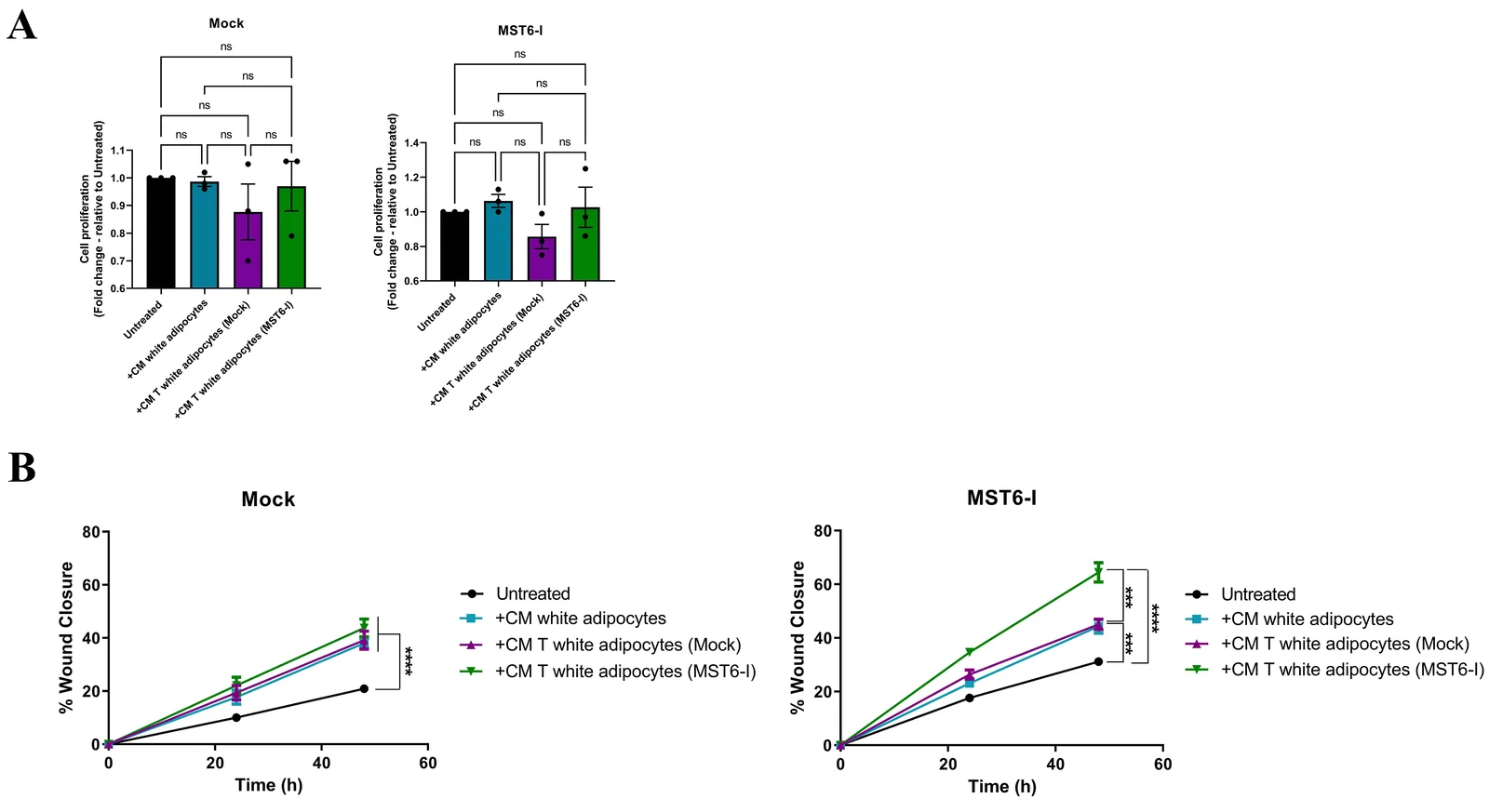
Adipocyte-derived secretome enhances the migration capacity of gastric cancer cells. (A) Proliferation capacity of Mock and MST6-I cells after exposure to the secretome/CM from white or transdifferentiated/beige adipocytes. One-way ANOVA was used for statistical analysis. (B) Migration capacity of Mock and MST6-I cells after exposure to the secretome/CM from white or transdifferentiated/beige adipocytes. Two-way ANOVA was used for statistical analysis. Biological replicates are represented as individual data points. ns: *P*-value > 0.05; ***: *P*-value ≤ 0.001; ****: *P*-value ≤ 0.0001. CM: Conditioned medium; ANOVA: analysis of variance.

Together, these results showed that exposure of MST6-I cells to secretomes from white and beige-like adipocytes promoted a metabolic shift towards increased fatty acid uptake and oxidation, increased mitochondrial number, and reduced cytoplasmic lipid accumulation, indicating enhanced fatty acid consumption. Furthermore, we observed increased migration in both Mock and MST6-I cells after treatment with secretomes from white or beige-like adipocytes, with MST6-I cells showing the most pronounced response. 

## DISCUSSION

The metabolic crosstalk between tumor cells and adipose tissue is recognized as a key driver of tumor growth, dissemination, and systemic metabolic dysfunctions, including cancer cachexia^[17,55-57]^. This metabolic syndrome, characterized by severe loss of body fat and skeletal muscle, is particularly concerning since cancer cachexia profoundly affects patients' quality of life and treatment outcomes^[29]^. Currently, management of cachexia is limited to treating the underlying conditions and providing nutritional support, underscoring the urgent need for novel therapeutic targets^[58]^. Given the proximity of the stomach to visceral fat depots, adipocytes may actively support gastric cancer progression through endocrine and paracrine mechanisms^[59-62]^. EVs have emerged as crucial messengers in tumor cell-adipocyte interplay, carrying bioactive molecules that reprogram the metabolism and phenotype of recipient cells^[5,6]^. Notably, tumor-derived EVs can induce substantial metabolic reprogramming of adipocytes, thereby contributing to the development and progression of cancer cachexia^[5,6]^. Importantly, we previously observed that gastric cancer cells expressing short *O*-glycans, such as the STn antigen, induce unintentional weight loss in mouse models, which was reversed after tumor removal^[37]^, underscoring the putative clinical implications of tumor glycosylation in tumor progression, particularly in cancer cachexia. Furthermore, these short *O*-glycans have also been detected in gastric cancer-derived EVs^[50,52]^. Despite growing recognition of the role of EVs in tumor-adipocyte crosstalk, the impact of tumor-associated glycosylation on this metabolic interplay remains largely unknown.

To address this gap, we investigated how short *O*-glycans, particularly the STn antigen, present in gastric cancer cells and EVs, modulate communication between gastric cancer cells and adipocytes. Our results revealed that STn-positive cells release more EVs than STn-negative cells, with no significant differences in size distribution. Several studies have shown that glycans, including glycosphingolipids^[63]^ and proteoglycans^[31,64-66]^ can modulate exosome biogenesis and release. Specifically, previous research demonstrated that cancer cells overexpressing STn secrete more EVs^[52,67]^. Furthermore, EVs were enriched for *O*-glycoproteins carrying the STn antigen compared with their parental cells, indicating selective packaging of STn-modified proteins into EVs and suggesting that STn might play a role in both EV release and cargo selection.

Notably, STn-positive EVs were internalized by white adipocytes in a time-dependent manner. On the other hand, STn-negative EVs were not internalized by adipocytes within 24 h of exposure. This preferential uptake likely enables STn-positive EVs to exert an effect on recipient adipocytes, in contrast to STn-negative EVs. Previous studies have shown that the surface glycans of EVs significantly influence their recognition and uptake by recipient cells, thereby modulating their biological effects^[31,32]^. Indeed, our results showed that STn-positive EVs induced pronounced reprogramming of white adipocytes toward a beige-like state. This transformation was evidenced by the upregulation of the browning genes *Ucp1* and *Prdm16*, activation of HSL (p-HSL^660^), and increased glycerol and free fatty acid release. Interestingly, *Ucp1* expression in adipocytes increased after exposure to STn-positive EVs, consistent with the trend toward enhanced internalization. These results suggest that the presence of STn, or the absence of more elongated *O*-glycans, in EVs is essential for EV internalization by adipocytes, thereby contributing to their browning phenotype. Previous studies in pancreatic and breast cancers have shown that tumor-derived EVs or direct tumor cell-adipocyte contact stimulate lipolysis in adipocytes, a response characterized by reduced triglyceride content, increased release of glycerol and free fatty acids, and HSL activation^[68-70]^. In breast cancer, the lipolytic response of adipocytes triggered by tumor-derived EVs was also associated with the transfer of cyclic adenosine monophosphate (cAMP)-dependent protein kinase A (PKA) from cancer cells to adipocytes^[70]^. Moreover, EVs from lung, gastric, and prostate cancer cells have been reported to increase intracellular cAMP levels and PKA activity in adipocytes^[20,71,72]^. Other studies noted that pancreatic cancer-derived exosomes carrying adrenomedullin activated the p38 and the Ras-dependent extracellular signal-regulated/mitogen-activated protein kinases (ERK/MAPK) signaling pathways in adipocytes, resulting in HSL phosphorylation and glycerol release^[73]^. Furthermore, EVs carry miRNAs (miR204-5p and miR-155) that have been implicated in increasing leptin and hypoxia-inducible factor 1α (HIF-1α) levels in adipocytes^[74]^, regulating lipolytic enzymes such as HSL and ATGL, reducing lipogenesis, and stimulating adipocyte browning by inducing *Ucp1* upregulation^[75]^. In line with these observations, our data suggest that STn-positive gastric cancer cells and EVs drive metabolic reprogramming of adipocytes, enhancing lipid mobilization and fostering a nutrient-rich environment that supports tumor growth. This remodeling of white adipocytes into beige-like adipocytes often results in global body weight loss, contributing to the cancer cachexia phenotype^[57]^. Importantly, several additional studies have shown that EV effects on adipocyte browning and lipolysis in the prostate^[72]^, breast^[74-76]^, gastric^[19]^, colorectal^[77]^, and lung cancers^[71,78-80]^, often involve the transfer of specific proteins, miRNAs, or hormones. Although these studies did not address glycosylation, STn is highly prevalent in these cancer types^[81]^, which may also contribute to the observed EV-mediated adipocyte browning. It is also worth noting that the secretome from STn-negative cells reduced ACC expression, increased p-HSL^660^ activation, and increased glycerol release. However, their derived EVs did not show the same effect, most likely because they were not internalized. Furthermore, other secretome components from STn-negative cells may also contribute to lipolytic activity in white adipocytes. Indeed, tumor cells can release interleukins (IL-6 and IL-1β), TNF-α, interferon gamma (IFN-γ), zinc-α2-glycoprotein (ZAG), or parathyroid hormone-related protein (PTHrP), which can promote adipocyte browning and lipolysis^[82-85]^.

Given the role of STn-positive gastric cancer cells and their derived EVs in remodeling adipocyte phenotype and metabolism, we next evaluated whether tumor cells use free fatty acids released by these adipocytes. Remarkably, we found that STn-positive cells exhibit increased free fatty acid uptake and display metabolic features consistent with enhanced fatty acid oxidation. Seahorse analysis revealed a compensatory shift toward glycolysis after etomoxir-mediated inhibition of fatty acid oxidation, indicating metabolic flexibility in STn-positive cells. Moreover, the increased fluorescence intensity of BODIPY-labeled fatty acids in these cells after etomoxir treatment further supports the reliance of STn-positive cells on fatty acids as an energy source. Importantly, these effects were not observed in STn-negative cells, highlighting a distinct metabolic profile between STn-positive and STn-negative gastric cancer cells. Previous studies in colon and breast cancer cells have reported that enhanced fatty acid oxidation correlates with 5' adenosine monophosphate-activated protein kinase (AMPK) activation, reduced ACC levels, and upregulation of carnitine palmitoyltransferase 1 (CPT1)^[86,87]^. Furthermore, the adipocyte-derived secretome stimulated expression of phosphatidylinositol transfer protein, cytoplasmic 1 (PITPNC1) in gastric cancer cells, thereby promoting expression of SREBP1, PPARγ, CD36, and CPT1B, facilitating fatty acid uptake and stimulating fatty acid oxidation in these cells^[60]^. Importantly, consistent with these reports, our transcriptomic analysis revealed increased expression of the protein kinase AMP-activated non-catalytic subunit beta 2 (PRKAB2), a regulatory subunit of AMPK^[88]^, in STn-positive cells after exposure to secretomes from white or beige-like adipocytes. This finding suggests a shift toward AMPK-driven oxidative metabolism, enabling STn-positive cells to efficiently use fatty acids as an energy source under metabolic stress and highlighting AMPK as a potential therapeutic target to mitigate adipocyte-mediated metabolic dysfunctions and disease progression in gastric cancer. Interestingly, inhibition of fatty acid oxidation with etomoxir has also been shown to redirect tumor cell energy production towards glucose metabolism^[89]^.

Building on these metabolic insights, we further explored how secretomes from white and beige-like adipocytes affect lipid metabolism pathways in tumor cells. We found that gastric cancer cells treated with adipocyte secretomes undergo metabolic reprogramming from lipogenesis to lipid remodeling, polyunsaturated fatty acid (PUFA) metabolism, and mitochondrial and peroxisomal β-oxidation. Untreated gastric cancer cells displayed higher expression of genes involved in fatty acid synthesis (*SCD5, FASN, SCD,* and* FADS1*), triglyceride metabolism (DGAT2), and lipogenesis regulation (*INSIG1 *and *SREBF1*), indicating endogenous lipid production and storage. Conversely, exposure of gastric cancer cells to secretomes from white or beige-like adipocytes promoted upregulation of phospholipases and lipid-remodeling enzymes (*AIG1, PLA2G1,* and* PLA2G4F*) and of genes related to PUFA oxidation (*MFSD2A, ACOX2, *and* CYP2C19*). Upregulation of AIG1^[90,91]^ and PLA2 family^[92-94]^ enzymes suggest increased hydrolysis of glycerophospholipids and lipid esters, thereby releasing free fatty acids that could sustain mitochondrial and peroxisomal β-oxidation. Furthermore, MFSD2A^[95]^, ACOX2^[96]^, and CYP2C19^[97,98]^ expression has been shown to support increased PUFA uptake and metabolism, as well as peroxisomal β-oxidation of long-chain fatty acids. Altogether, our findings indicate that the adipocyte-derived secretome reprograms gastric cancer cell metabolism, favoring lipid uptake and oxidation, potentially driving tumor growth and progression. Notably, we also detected Cav-1 upregulation in STn-positive cells after exposure to the secretome from white or beige-like adipocytes. Cav-1 is known to regulate fatty acid uptake by modulating the localization and function of the fatty acid translocase (FAT/CD36)^[99,100]^. Adipocytes have also been shown to promote fatty acid uptake and metastatic potential in ovarian cancer cells. By upregulating fatty acid–binding protein 4 (FABP4)^[101-103]^ or CD36^[104]^ in tumor cells, adipocytes could facilitate the uptake and accumulation of long-chain fatty acids. In addition, depriving ovarian cancer cells of adipocyte-derived fatty acids by CD36 inhibitors reduced their adhesion, migration, and invasion capacities^[104]^. Moreover, activation of the AXL–CAV1–β-catenin signaling axis has been implicated in the invasive potential of melanoma cells^[105]^. Interestingly, our results further showed that the adipocyte-derived secretome (from white or beige-like adipocytes) also increased the migration capacity of gastric cancer cells, particularly when STn-positive cells were treated with secretome from beige-like adipocytes. Several other types of cancer have already shown that exposure to adipocytes can enhance the motility of breast^[22,106,107]^, melanoma^[21,108]^, colorectal^[109]^, pancreatic^[110,111]^, prostate^[112]^, hepatocellular^[24]^, and nasopharyngeal cancers^[113]^ cells. This pro-migratory phenotype has been linked to adipokines (IL-8 and IL-1β) and fatty acids released by adipocytes^[22,101,111]^, increased HIF-1α activity^[106,112]^, and activation of several signaling pathways^[114,115]^, highlighting how the secretome and EVs derived from adipocytes not only supply metabolic substrates but also promote malignant features in tumor cells. Together, our results suggest that the adipocyte secretome may facilitate tumor progression by enhancing gastric cancer cell migration. In addition, increased fatty acid uptake by STn-positive cells may result from Cav-1 upregulation, which could regulate CD36 localization and function, as reported in other cell types. Although CD36 did not appear upregulated in our RNA-seq analysis, as Cav-1 did, we cannot exclude that the Cav-1/CD36 pathway may represent another therapeutic target to inhibit fatty acid internalization by tumor cells and ultimately impair adipose tissue consumption.

In line with this metabolic remodeling, we also observed an increase in mitochondrial number and upregulation of lipid remodeling enzymes, including patatin-like phospholipase domain-containing 8 (PNPLA8) and ACAD11, in STn-positive gastric cancer cells after exposure to the secretome from white or beige-like adipocytes. This response aligns with previous findings in breast and melanoma cancer models, in which adipocyte-derived EVs, leptin, and fatty acids promote mitochondrial reprogramming and fatty acid oxidation in tumor cells^[22,87,108,116]^. Furthermore, treatment of tumor cells with exogenous leptin enhanced lipid consumption for energy production^[117,118]^. PNPLA8, also known as iPLA2γ, is a mitochondrial and peroxisomal phospholipase that could promote the production of unsaturated fatty acids and eicosanoids^[119]^. Its overexpression was associated with phospholipid metabolic reprogramming, cell viability, and migration in breast cancer^[119]^, and with metastases and poor prognosis in patients with colorectal cancer^[120]^. Furthermore, H_2_O_2_-mediated activation of iPLA2γ could promote its interaction with mitochondrial carriers such as UCPs, thereby preventing oxidative stress^[121,122]^. ACAD11, a p53-regulated mitochondrial acyl-CoA dehydrogenase, supports tumor cell survival under metabolic stress^[123,124]^. Therefore, the observed upregulation of genes involved in mitochondrial function and lipid remodeling in our study may reflect an adaptive response that enhances mitochondrial fatty acid mobilization, thereby contributing to the metabolic flexibility of STn-positive gastric cancer cells and enabling them to better adapt and thrive in diverse metabolic environments.

Given the role of EVs in mediating tumor-adipocyte communication, we assessed how adipocytes influence the biogenesis of tumor-derived EVs. Notably, we observed an increase in endosomes in STn-positive cells after exposure to the secretome from beige-like adipocytes. Similarly, treating breast cancer cells with leptin increased exosome release by upregulating Tsg101, an important subunit of the endosomal sorting complex required for transport I (ESCRT-I)^[125]^. This suggests that adipocyte-derived factors in the adipocyte secretome may enhance EV biogenesis in cancer cells, potentially amplifying reciprocal communication between tumor cells and adipocytes. Therefore, targeting EV biogenesis, release, or uptake by recipient cells may represent a promising therapeutic approach to disrupt this tumor-adipocyte metabolic crosstalk and impair tumor progression. Indeed, pharmacological or genetic inhibition of EV release (using GW4869^[126]^, Rab27A knockdown^[71]^, inhibition of HSP70/HSP90-dependent EV release with omeprazole^[127]^ or atractylenolide I treatment^[128]^) has been shown to inhibit adipocyte transdifferentiation and lipolysis and to attenuate weight loss in mouse models of lung and colon cancer, supporting the therapeutic potential of EV-targeted therapies to disrupt the metabolic interplay between tumor cells and adipocytes that fuels tumor progression and cancer cachexia. Recent evidence indicates that tissue-resident macrophages clear locally released EVs and those entering from circulation, thereby influencing circulating EV levels^[129]^. Importantly, the presence of STn in tumor cells and potentially in their EVs may protect them from immune clearance^[130]^. Nevertheless, these generic strategies might have substantial side effects in cancer patients. Therefore, targeting STn-positive EVs in circulation to block tumor-adipocyte crosstalk might be a promising approach to tackle cancer cachexia.

### Limitations of the study

This study provides important insights into the role of STn-positive EVs in mediating metabolic crosstalk between gastric cancer cells and adipocytes. However, several limitations should be considered. This study was based on *in vitro* observations. Although *in vitro* systems offer control over experimental conditions, they do not fully replicate the complexity of tumor-host interactions in a physiological context. Using primary adipocytes, additional cancer cell lines, or patient-derived gastric organoids would enhance the translational relevance of our results. Furthermore, given that STn is highly synthesized across multiple cancer types, it would be important to validate whether STn-associated effects on adipocyte browning extend beyond gastric cancer. Our results suggest that STn in both cells and EVs serves as a key mediator of adipocyte remodeling. Nevertheless, STn-positive EVs lack elongated *O*-glycans. Indeed, STn-negative EVs that display elongated *O*-glycans were not internalized by adipocytes and did not induce the browning and lipolytic responses observed with STn-positive EVs. Future experiments addressing other glycosylation traits and aiming to identify EV protein carriers could reveal additional mechanisms with therapeutic implications for targeting tumor-adipocyte communication.

### Conclusions

Our results show that EV glycosylation, specifically STn, plays a crucial role in intercellular communication between gastric cancer cells and adipocytes. STn-positive EVs induced browning and lipolysis in white adipocytes, resulting in the release of glycerol and fatty acids. In turn, fatty acids released by adipocytes were internalized by STn-positive gastric cancer cells, reprogramming their metabolism toward fatty acid oxidation, accompanied by increased mitochondrial number and enhanced tumor cell migration. We propose that tumor-associated glycosylation, particularly STn in gastric cancer cells and EVs, enhances EV uptake by adipocytes, thereby initiating a reciprocal metabolic reprogramming cycle that drives tumor progression. Disrupting this communication axis may offer a promising strategy to disrupt the metabolic co-dependency between tumor cells and adipocytes, ultimately improving clinical outcomes for gastric cancer patients and providing new insights into the management of cancer cachexia. 

## DECLARATIONS

### Availability of data and materials

RNA-Seq data are available at the European Nucleotide Archive (ENA), under the Project ID PRJEB105016. Metabolomics data are available at the NIH Common Fund's National Metabolomics Data Repository (NMDR) website and the Metabolomics Workbench (https://www.metabolomicsworkbench.org), where they have been assigned Project ID PR002875. The data can be accessed directly via its Project DOI: (https://doi.org/http://dx.doi.org/10.21228/M81V97). This work is supported by Metabolomics Workbench/National Metabolomics Data Repository (NMDR) (U2C-DK119886) and Common Fund Data Ecosystem (CFDE) (OT2-OD030544).

### AI and AI-assisted tools Statement

During the preparation of this manuscript, the AI tool Grammarly (v1.2.228.1813) was used solely to improve spelling, grammar, clarity, and readability during manuscript preparation. The tool did not influence the study design, data collection, analysis, interpretation, or the scientific content of the work. All authors take full responsibility for the accuracy, integrity, and final content of the manuscript.

### Copyright

© The Author(s) 2026.
