# Supplementary material for: Glycosylated extracellular vesicles drive a metabolic interplay between gastric cancer cells and adipocytes
Source: Extracell Vesicles Circ Nucl Acids. 2026 Jul 6;7(3):1056–83. doi: 10.20517/evcna.2026.16 (PMC13673474; doi:10.20517/evcna.2026.16)
Supplement: Supplementary file 1 [file evcna-7-3-1056-SupplementaryMaterials.zip › evcna7016-SupplementaryMaterials.pdf]

**DOI**: 10.20517/2574-1209.xxxx.xx

**Extracellular Vesicles and Circulating Nucleic Acids**

**Supplementary Materials**

# Glycosylated extracellular vesicles drive a metabolic interplay between gastric cancer cells and adipocytes

**Cátia C Ramos[1,2,3], Oihane E Albóniga[4], Adriana R Rodrigues[1,5,6], Clara Garcia-Vallicrosa[7], Joana Peixoto[1], Rui Fernandes[1], Bruno Cavadas[1], Alexandra M Gouveia[1,5], Celso A Reis[1,2,3,8], Juan M Falcon-Pérez[4,7,9], Daniela Freitas[1,2,8]**

[1]i3S - Instituto de Investigação e Inovação em Saúde, Universidade do Porto, Porto 4200, Portugal.

[2]IPATIMUP - Instituto de Patologia e Imunologia Molecular da Universidade do Porto, Porto 4200, Portugal.

[3]ICBAS - Instituto de Ciências Biomédicas Abel Salazar, Universidade do Porto, Porto 4050, Portugal.

[4]Metabolomics Platform, CICbioGUNE-BRTA, Derio 48160, Spain.

[5]Departamento de Biomedicina, Unidade de Biologia Experimental, Faculdade de Medicina do Porto, Porto 4200, Portugal.

[6]Faculdade de Ciências da Nutrição e Alimentação, Universidade do Porto, Porto 4200, Portugal.

[7]Exosomes Laboratory, CIC bioGUNE-BRTA, CIBERehd, Derio 48160, Spain.

[8]Faculdade de Medicina do Porto, Porto 4200, Portugal.

[9]IKERBASQUE Research Foundation, Bilbao 48009, Spain.

**Correspondence to:** Dr. Daniela Freitas, i3S - Institute for Research and Innovation in Health, University of Porto, Porto 4200, Portugal. E-mail: dfreitas@i3s.up.pt

© The Author(s) 2020. Open Access This article is licensed under a Creative Commons Attribution 4.0 International License (https://creativecommons.org/licenses/by/4.0/), which permits unrestricted use, sharing, adaptation, distribution and reproduction in any medium or format, for any purpose, even commercially, as long as you give appropriate credit to the original author(s) and the source, provide a link to the Creative Commons license, and indicate if changes were made.

OAE Publishing Inc.

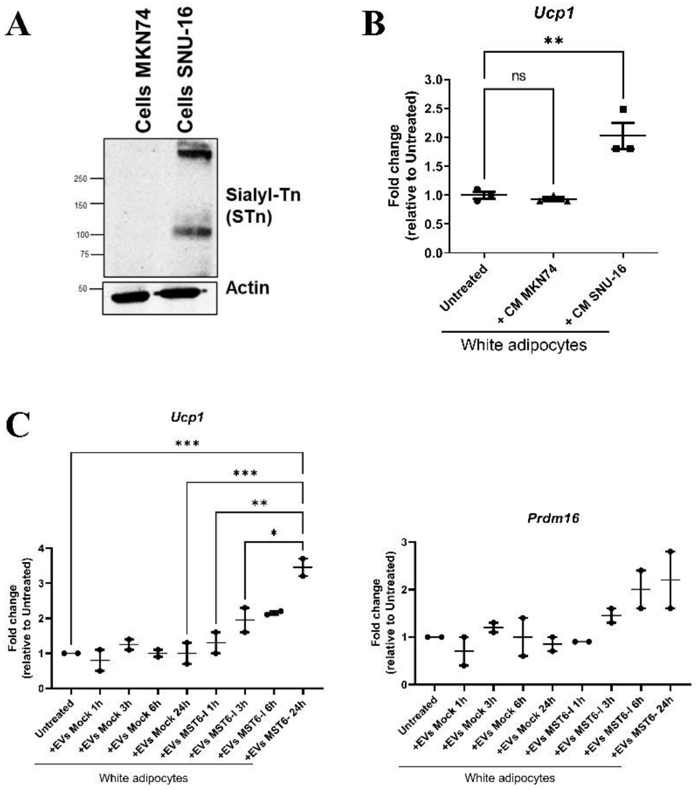


**Supplementary Figure 1.** Secretome and EVs derived from STn-positive gastric cancer cells induce metabolic reprogramming in 3T3-L1 adipocytes. (A) Western blot analysis of the STn antigen in SNU-16 and MKN74 cells. One biological replicate was performed. (B) RT-qPCR analysis of *Ucp1* expression in adipocytes treated with the secretome/conditioned medium (CM) of gastric cancer cells that express (SNU-16) or do not express (MKN74) the STn antigen. Results are shown as mean ± SEM. One-way ANOVA was used for statistical analysis (C) RT-qPCR analysis of *Ucp1* (left panel) and *Prdm16* (right panel) expression in adipocytes exposed to 5 µg of Mock and MST6-I EVs at different time points (1 hour, 3 hours, 6 hours, and 24 hours). Results are shown as mean ± SEM. One-way ANOVA was used for statistical analysis; unless otherwise indicated, differences were not significant (ns). Biological replicates are represented as individual data points. ns: p-value > 0.05; *: p-value ≤ 0.05; **: p-value ≤ 0.01; ***: p-value ≤ 0.001.

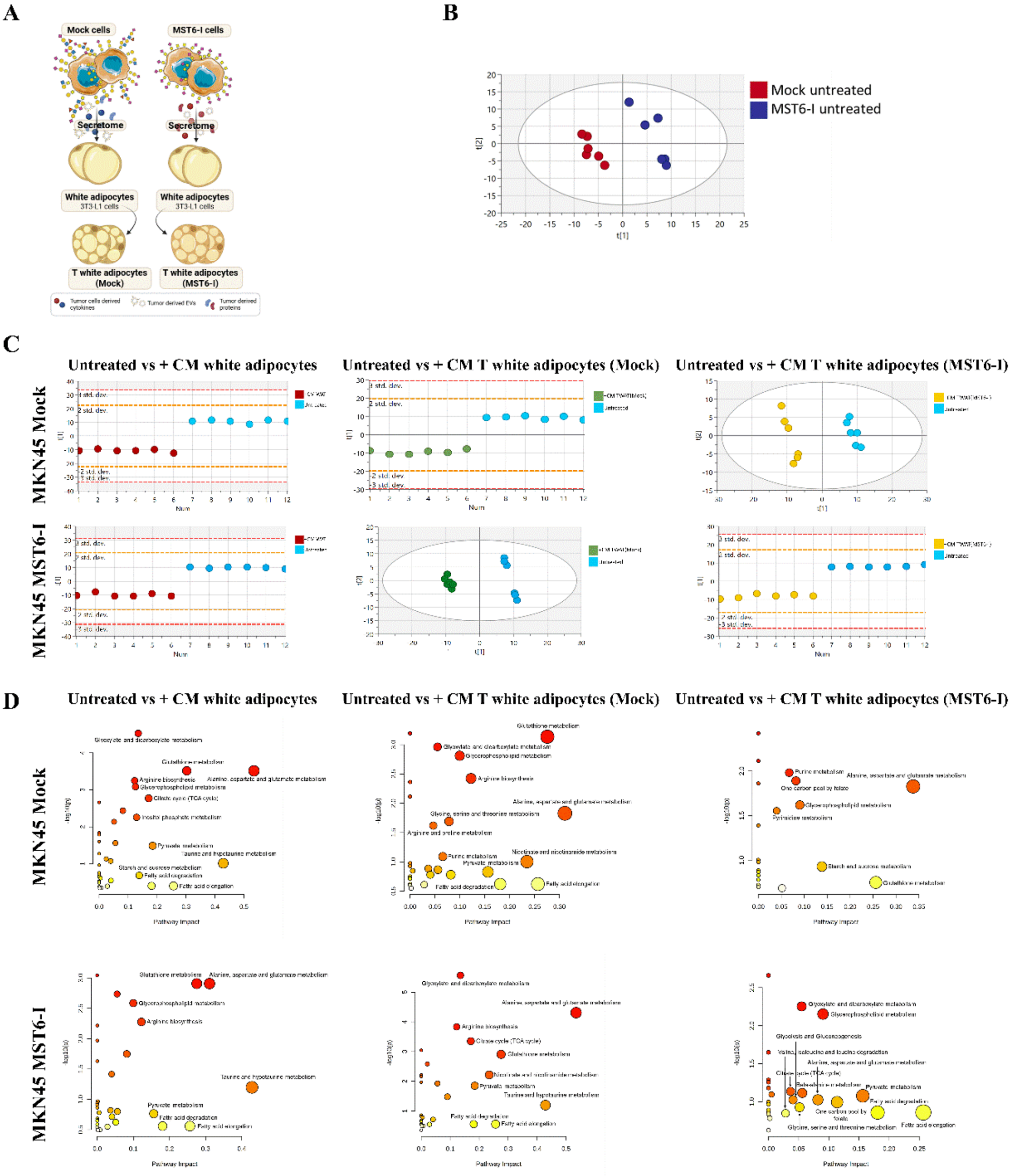


**Supplementary Figure 2.** Pathway analysis of the metabolic reprogramming of gastric cancer cells after treatment with the adipocyte-derived secretome. (A) Schematic representation of the experimental workflow illustrating the generation of transdifferentiated (beige-like) adipocytes by treating cells with secretome/conditioned medium (CM) from Mock (TWAT(Mock)) or MST6-I (TWAT(MST6-I)) cells. Created with BioRender.com. (B) Partial least squares-discriminant analysis (PLS-DA) score plots of untreated Mock and MST6-I cells (ESI+ mode, Pareto scaling). Significant metabolic features were identified with VIP > 1 and |p(corr)| > 0.85. (C) PLS-DA score plots of Mock and MST6-I cells treated with secretome/CM from different adipocyte

types compared with untreated cells (ESI+ mode, Pareto scaling). Significant metabolic features were identified with VIP > 1 and |p(corr)| > 0.85. (D) Pathway enrichment analysis indicating pathway impact (circle size) and statistical significance (color intensity), with more intense red indicating a lower p-value.

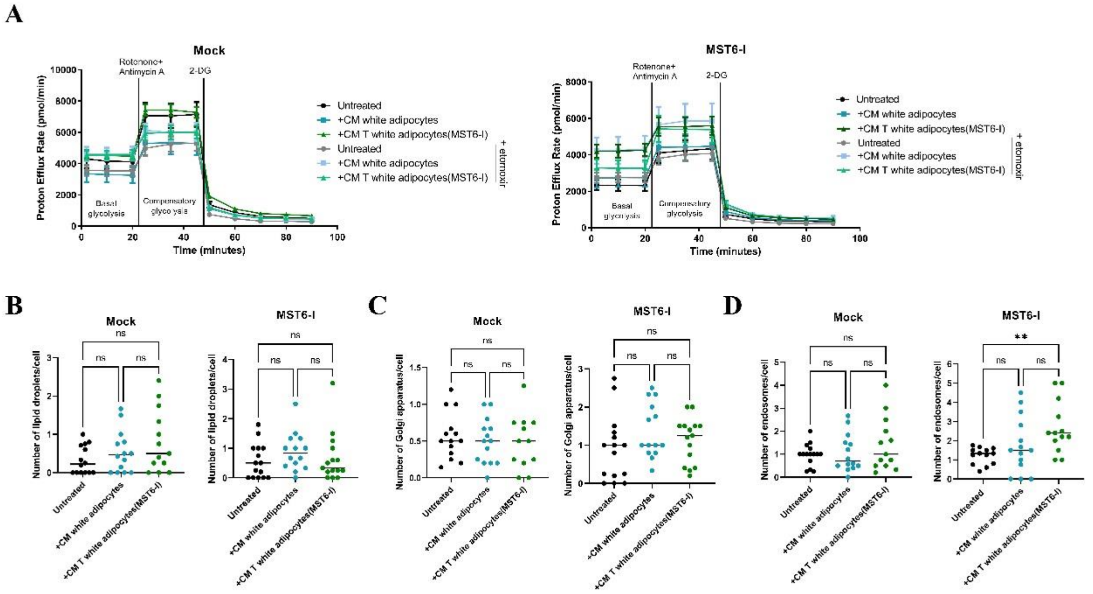


**Supplementary Figure 3.** The adipocyte-derived secretome induces a metabolic shift and organelle remodeling in STn-positive gastric cancer cells. (A) Seahorse analysis of the Proton Efflux Rate (PER) in Mock (left panel) and MST6-I (right panel) cells, untreated or treated with secretome/conditioned medium (CM) from white and transdifferentiated/beige-like adipocytes (TWAT(MST6-I)), with or without etomoxir. (B-D) Quantification of the number of lipid droplets (B), Golgi apparatus (C), and endosomes (D) per cell in Mock and MST6-I cells after treatment with secretome/CM from white or transdifferentiated/beige-like (TWAT(MST6-I)) adipocytes, based on TEM imaging. Results are shown as mean ± SEM, with statistical significance determined by One-way ANOVA. ns: p-value > 0.05; **: p-value ≤ 0.01.
